# Screen time and health issues in Chinese school-aged children and adolescents: a systematic review and meta-analysis

**DOI:** 10.1186/s12889-022-13155-3

**Published:** 2022-04-22

**Authors:** Youjie Zhang, Shun Tian, Dan Zou, Hengyan Zhang, Chen-Wei Pan

**Affiliations:** School of Public Health, Medical College of Soochow University, 199 Ren Ai Road, 215123 Suzhou, Jiangsu China

**Keywords:** Screen time, Child health, Chinese, Child, Adolescent

## Abstract

**Backgrounds:**

Many literature reviews summarized relationships between screen time and child health, but they only included a few studies conducted in Chinese children and adolescents. The potential influence of screen time may vary by social context. The current systematic review and meta-analysis aimed to evaluate relationships between screen time and health issues among Chinese school-aged children and adolescents.

**Methods:**

Peer-reviewed articles written in Chinese and English were retrieved from CNKI, Wanfang, PubMed, Embase, and Web of Science from inception to June 2020. The Downs & Black checklist was applied to assess study quality. Meta analyses used random effect models and mixed effects model to calculate pooled adjusted odds ratios and 95% confidence intervals. Heterogeneity, sensitivity, and publication bias were assessed using Q and I^2^ statistics, “one-study removed” analysis, the funnel plot, trim and fill analysis, and classical fail-safe N, respectively.

**Results:**

In total, we identified 252 articles reporting 268 studies with unique samples. These studies investigated relationships between screen time and health issues of adiposity, myopia, psycho-behavioral problems, poor academic performance, cardiometabolic disease risks, sleep disorder, poor physical fitness, musculoskeletal injury, sub-health, and miscellaneous issues of height and pubertal growth, injury, sick leave, and respiratory symptoms. Proportions of studies reporting positive relationships with screen time were lowest in adiposity (50.6%) and higher in myopia (59.2%) and psycho-behavioral problems (81.8%). Other health issues were examined in 10 or less studies, all of which had more than half showing positive relationships. The pooled odds ratio from 19 studies comparing health risks with the screen time cutoff of 2 hours per day was 1.40 (95% CI: 1.31 to 1.50, *I*^2^ = 85.9%). The pooled effect size was 1.29 (95% CI: 1.20 to 1.39) after trimming 7 studies for publication bias adjustments.

**Conclusions:**

Findings exclusively generated from Chinese school-aged children and adolescents resonate those mainly from western countries. Evidence suggests that higher levels of screen time are related with greater risks of various health issues, although the relationships appear to be weak and intertwined with other confounding factors. Future studies need to investigate health-specific dose effects and mechanisms of screen time.

**Supplementary information:**

The online version contains supplementary material available at 10.1186/s12889-022-13155-3.

## Background

Electronic devices have become daily essentials in the modern days even for the young. Children and adolescents spend more time on screen-based activities than ever before [1, 2]. Concerns over the adverse effects of screen time have increased. Several authoritative organizations have published guidelines for professionals and families to manage screen time for children and adolescents [3–7]. The well-known recommendation is to have no more than 2 h of recreational screen time per day [7]. However, evidence on the unfavorable relationships between excessive screen time and various health risks remain limited due to inconsistent research findings and unclear threshold effects [8]. Current evidence on the potential impact of screen time has been majorly summarized from studies conducted in high-income Western countries [9, 10]. Evidence from other regions may make additional contributions to the existing knowledge base [11, 12].

In China, children and adolescents’ screen time have increased significantly [13]. According to a 2016 national report, 36.8% of school-aged children from 4 to 12 grades spent more than 2 h of screen time per day [14]. Considering the concurrent rises of pediatric obesity, myopia, and mental health problems among Chinese youth [15–17], a considerable number of studies examined the potential health impact of screen time. But these studies are not captured in existing reviews. Chinese children and adolescents live in an environment where is culturally, socially, and physically different from the West. Two significant differences make the investigation on Chinese youth’s screen time unique. One is the prolonged overall sedentary time; the other is the relatively isolated cyberspace. One widely recognized adverse health feature of screen time is being sedentary. An international study found that Chinese 9- to 11-year-olds had the highest presence of “sitters” characterized by high sedentary time and low physical activity in both boys and girls as compared to the averages of children from 12 nations (boys: 56% vs. 27%, girls: 59% vs. 32%) [18]. This is largely due to the nationwide academic devotions [19]. The influence of screen time on sedentism-related health issues among a population with prolonged sedentary time would add additional insights. In terms of the influence related to content exposure, Chinese children and adolescents live in a digital environment which is separated from the rest of the world due to the language barrier and internet censorship. It would be interesting to examine whether similar relationships between screen time and health issues exist among Chinese youth as compared to counterparts living in western countries.

Systematic reviews are unbiased comprehensive syntheses of scholarly investigations on well-defined research questions, which also enable meta-analyses of statistical results of different studies. On the topic of screen time-related health influences, many systematic reviews have been conducted. For example, Stiglic and Viner identified 13 systematic reviews, published till 2018, on the well-being effects of screen time in children and adolescents [8]. Thereafter, at least seven systematic reviews have expanded on this topic [20–26]. Health issues addressed in these reviews included body composition, dietary intake, mental health, cardiovascular risks, fitness, sleep, pain, asthma, myopia, and language skills. Among these health issues, adiposity and depressive symptoms have shown relatively stronger evidence for associations with screen time, while evidence on other health issues is insufficient. Despite the plethora of reviews, only a few studies conducted in China were included, which is far less than the number of studies found in our preliminary search.

Therefore, with the purpose of addressing this evidence gap, the current systematic review aimed to (1) identify studies examining relationships between screen time and health issues among Chinese school-aged children and adolescents from both Chinese and English literature databases, (2) summarize health issues that showed associations with screen time, and (3) use available data to quantify the relationship between screen time and child health. Findings gather in this review can supplement the existing evidence bank from a population-specific perspective and offer insights for advancing screen time-related health research.

## Methods

### Search strategy

Articles written in Chinese were retrieved from the China National Knowledge Infrastructure (CNKI) and Wanfang Data platforms which host the most comprehensive lists of Chinese academic journals and offer the largest access to full-text Chinese journal articles. Articles written in English were searched from PubMed, Embase, and Web of Science databases. Search terms were combinations of terms related to school-aged children and adolescents, screen-based behaviors, and geographic locations of mainland China, Hongkong, Macau and Taiwan (Table A.1). No limits were imposed on the publication date, the final search was conducted on June 1, 2020.

### Inclusion and exclusion criteria

Eligible studies were identified according to the pre-specified inclusion and exclusion criteria following the Population, Intervention, Comparison, Outcome, Study design (PICOS) framework [27]. The inclusion criteria for study selection were (1) peer-reviewed articles written in Chinese or English, (2) study participants were children and adolescents with age ranges or mean ages between 6 and 18 years, or enrolled in grade 1 through grade 12, (3) observational or experimental study design, (4) study participants were not diagnosed with phycological or physical diseases at baseline, (5) studies reported relationships between screen time and certain health indicators. Screen time refers to the time spent on screen-based behaviors [28]. The current review did not confine to specific health outcomes and followed the World Health Organization’s definition of health that is “a state of complete physical, mental, and social well-being and not merely the absence of disease or infirmity” [29]. Exclusive criteria were (1) conference abstracts and non-original research articles, (2) study participants were diagnosed with diseases at baseline, (3) repetitive publications, (4) studies that did not report relationships between screen time and health indicators, (5) cross-sectional surveys reporting associations between screen time and internet addiction if measures of internet addiction included excessive screen time, (6) observational studies with sample sizes less than 300 and experimental studies with sample sizes less than 30 [30].

### Study selection and data extraction

Entries identified from each bibliographic database were imported to EndNote™ 20. Duplicates were removed using the deduplication function of the program, and then manually checked by two reviewers (Y. Z. and S. T.). Titles and abstracts of the remaining entries were screened by the two reviewers based on the pre-specified eligibility criteria. Results from the preliminary screening were compared, and inconsistencies were discussed and resolved. Full-text articles were obtained after the preliminary screening. The two reviewers examined the full-texts independently and resolved discrepancies through intensive discussion. Data extraction was performed by one reviewer (S.T., D.Z. or H. Z.) and checked by the other (Y. Z.). Data extraction table included following information: the first author’s name, year of publication, journal, language written, location, research design, age range, sample size, types of screen-based behaviors, health issues, adjusted covariates, and main results (Table A.2).

### Quality assessment

Study quality assessment was performed using the Downs & Black checklist [31]. The checklist showed high reliability and validity, and was applied in several systematic reviews of health behaviors [30, 32, 33]. The checklist can add up to a maximum score of 28 from 27 items assessing reporting quality, external validity of sample representativeness, internal validity of measurement and analytical biases, and selection bias. The item of confounder adjustments has a maximum of 2 points, 1 point for the adjustment of sociodemographic variables and the other for the adjustments of bio-behavioral factors. Two trained research assistants (D. Z. and H. Z.) assessed the included studies independently, and a third researcher (Y.Z.) compared and resolved discrepancies. The study quality scores were evaluated in three categories based on percentages of scores attained from the applicable items of corresponding study design: high (≥ 70%), moderate (50-69.9%), and low (< 50%) [32].

### Data analysis

Summative syntheses of the included studies include classification and narrative integration of categories of health issues, study design, and relationships between screen time and health indicators. Data extracted for meta-analysis included adjusted odds ratios and 95% confidence intervals from studies analyzed health risks associated with exceeding versus within 2 h of daily screen time using multiple logistic regression models. We did not extract linear regression results due to between-study heterogeneity in terms of screen time and outcome measures. Meta-analyses were performed using the Comprehensive Meta Analysis Version 3.3.070. Heterogeneity of pooled effect sizes was assessed by Q and I^2^ statistics. A significant Q statistic and I^2^ > 50% indicated a substantial heterogeneity and the selection of the random effect model [34]. Sensitivity of the meta-analysis was performed using the “one-study removed” analysis. Assessments of publication bias included asymmetric examination of the funnel plot, Duval and Tweedie’s trim and fill analysis and the Classical fail-safe N (Duval & Tweedie, 2000). In addition, a mixed effects analysis was applied to generate subgroup effects by health issues using random effects models and combine effects from subgroups to yield an overall effect using a fixed effect model.

## Results

### Search results

**Fig. 1:**
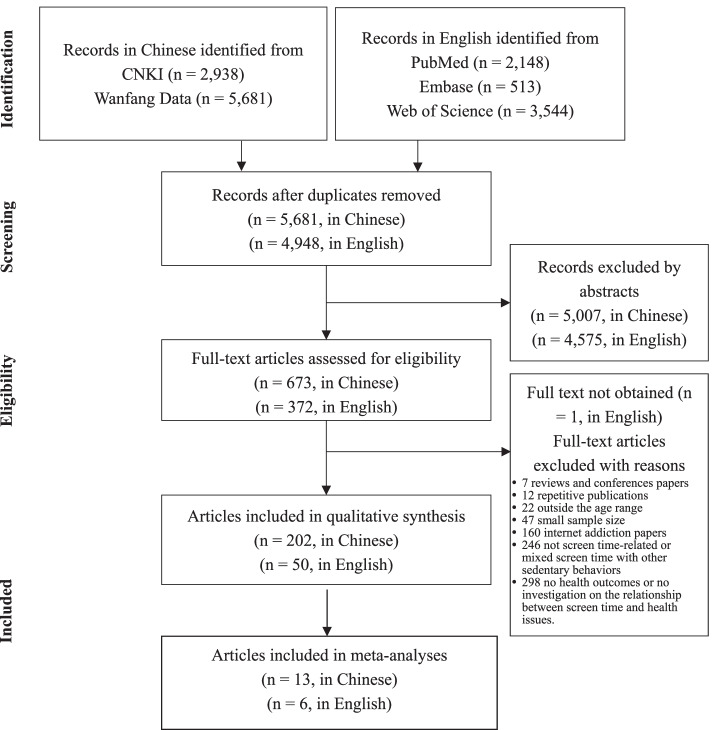
The PRISMA flow diagram

An overview of the record retrieval and selection process is shown in Fig. 1. A total number of 252 articles were retained for summative syntheses and data from 19 articles were pooled for meta-analyses. The included articles were published between 1999 and 2020, 62.3% were published in 2015 and after, 80.2% were written in Chinese, and 92.5% were conducted in mainland China. These articles reported original research findings regarding relationships between screen time and health issues of adiposity, myopia, psycho-behavioral problems, cardiometabolic disease risks, poor academic performance, sleep disorder, poor physical fitness, musculoskeletal injury, physical and mental sub-health, and a group of miscellaneous issues related to height or pubertal growth, injury, etc. According to the health issues, 268 studies with independent samples were identified, nearly 90% of which were in a cross-sectional design (Table 1). The percentages of study quality classification are 8.6% (high), 57.5% (moderate), and 34.0% (low). Detailed study quality classification by health issues is shown in Table 2.

**Table 1:** Characteristics of eligible articles and identified studies

Characteristics	n	Percentage
Total number of articles	252	
Language written		
Chinese	202	80.2
English	50	19.8
Location		
Mainland China	233	92.5
Hong Kong, Macau, or Taiwan	19	7.5
Year of publication		
2015 ∼2020	157	62.3
2010 ∼ 2014	66	26.2
2005 ∼ 2009	21	8.3
1999 ∼ 2004	8	3.2
Total number of studies	268	
Health issue		
Adiposity	89	33.2
Myopia	76	28.4
Psychological or behavioral problems	44	16.4
Cardiometabolic disease risks	10	3.7
Poor academic performance	9	3.4
Sleep disorder	9	3.4
Poor physical fitness	8	3.0
Musculoskeletal injury	8	3.0
Physical and mental sub-health	6	2.2
Miscellaneous issues^a^	9	3.4
Research design		
Cross-sectional	240	89.6
Case-control	14	5.2
Longitudinal	12	4.5
Experimental	2	0.7

^a^ miscellaneous issues included height growth (*k* = 2), puberty timing (*k* = 3), respiratory symptoms (*k* = 2), sick leave (*k* = 1), and injury (*k* = 1)

**Table 2:** Summary of study quality classification by health issues

Health issues, *k*	All	Study quality classification		
		High	Moderate	Low
Adiposity	89	9	52	28
Myopia	76	9	44	23
Psycho-behavioral problems	44	1	26	17
Cardiometabolic issues	10	4	6	0
Poor academic performance	9	0	4	5
Sleep disorder	9	0	6	3
Poor physical fitness	8	0	7	1
Musculoskeletal injury	8	0	4	4
Physical and mental sub-health	6	0	5	1
Miscellaneous health issues	9	0	0	9
Total (%)	268	23 (8.6)	154 (57.5)	91 (34.0)

### Summary of study findings

Among the 268 unique studies, the numbers and percentages reporting positive, negative, insignificant, and inconsistent relationships between screen time and health risks were 169 (63.1%), 1 (0.3%), 35 (13.1%), and 63 (23.5%). Among the top-three most studied health issues, proportions of studies reporting positive relationships with screen time were lowest in adiposity (50.6%) and higher in myopia (59.2%) and psycho-behavioral problems (81.8%). Each of the other seven health issues had no more than 10 studies, and all had half or more showing positive relationships. By research design, case-control studies had a highest proportion (13 out of 14) showing positive relationships between screen time and health risks, following cross-sectional studies (62.1%), longitudinal studies (6 out of 12), and intervention studies (1 out of 2). Counts of study findings by health issues and research design are shown in Table 3.

**Table 3:** Summary of relationships between screen time and health risks by health issues and study design, and lower ends of screen-time cutoffs with significantly raised risks

Health issue	Research design	All	Relationship type^a^				Lower ends of significant cutoffs^b^ (*k*)
			+	-	0	?	
Adiposity	Intervention	1	0	0	1	0	TV: ≤1 h/d (5), ≤ 1.5 h/d (1), ≤2 h/d (4); C: ≤1 h/d (1), ≤2 h/d (2); G: ≤2 h/d (1); ST: ≤1 h/d (1), ≤2 h/d (7)
	Longitudinal	4	2	0	0	2	
	Case-control	8	7	0	1	0	
	Cross-sectional	76	36	0	15	25	
	Total	89	45	0	17	27	
Myopia,	Longitudinal	3	0	0	2	1	TV: ≤1 h/d (2), ≤2 h/d (1); C ≤ 1 h/time (1), ≤1 h/d (1), ≤2 h/d (1), ≤3 h/d (1); I: ≤ 2 h/d; non-TV ST: ≤1 h/d (1), ≤3 h/d (1); ST: 0 h/d (1), ≤ 0.5 h/d (2), ≤1 h/d (2), ≤2 h/d (2); ≤3 h/d (2).
	Case-control	4	4	0	0	0	
	Cross-sectional	69	41	1	10	17	
	Total	76	45	1	12	18	
Psycho-behavioral problems,	Intervention	1	1	0	0	0	TV: ≤1 h/d (2); I: entertainment 0 h/week (1), chatting 0 h/week (0), ≤ 10 h/week (1); G: ≤2 h/time (1); M: ≤20 min/d (1); ≤1 h/weekday (1), ≤1 h/weekend day (1); ST: ≤2 h/weekday (1), ≤2 h/weekend day (1), ≤2 h/d (5).
	Longitudinal	2	2	0	0	0	
	Cross-sectional	41	34	0	2	5	
	Total	44	37	0	2	5	
Cardio-metabolic disease risks	Case-control	1	1	0	0	0	TV: < 14 h/week (1); C: < 7 h/week (1); M: ≤3.5 h/week (1), ≤ 6 h/week (1); ST: ≤1 h/d (1), ≤2 h/d (3).
	Cross-sectional	9	5	0	2	2	
	Total	10	6	0	2	2	
Poor academic performance	Longitudinal	3	2	0	0	1	M: < 1 h/weekday (1), < 1 h/weekend day (1); non-TV ST: ≤1 h/d (1).
	Cross-sectional	6	6	0	0	0	
	Total	9	8	0	0	1	
Sleep disorders	Cross-sectional	9	7	0	0	2	TV: ≤2 h/weekday (1), ≤2 h/weekend day (1), ≤2 h/bedtime (1); C ≤ 1 h/d (1).
Poor physical fitness	Cross-sectional	8	5	0	0	3	ST ≤ 2 h/d (3), ≤2 h/weekday (1), ≤2 h/weekend day (1).
Musculoskeletal injuries	Cross-sectional	8	6	0	0	2	C ≤ 4 h/d (1); M: ≤4 h/d (1); ST: ≤1040 h (1).
Sub-health	Cross-sectional	6	6	0	0	0	I: ≤1 h/d (2), <4 h/d (1); ST: ≤2 h/weekday (1), ≤2 h/weekend day (1)
Miscellaneous issues	Case-control	1	1	0	0	0	TV: <1 h/d (1)
	Cross-sectional	8	4	0	2	2	
	Total	9	5	0	2	2	

*h/d* hours per day, *h/week* hours per week, *C* computer, *M* mobile phone, *I* internet, *G* electronic games, *ST* screen time, *non-TV ST* screen time excluding TV watching. The inconsistent cutoffs of “<” and “≤” were unified as “≤”

^a^ Relationship types: study findings showing positive (+), negative (-), null (0), and inconsistent (?) relationships between screen time and health risks

^b^ Lower ends of screen time cutoffs that associated with significantly reduced health risks when compared

Health issues without insignificant or inverse relationships with screen time were poor academic performance, sleep disorders, poor physical fitness, musculoskeletal injuries, and sub-health. Inconsistent findings within studies were related to sample characteristics (sex, age, area of residence, etc.), device types (TV, computer, electronic games), purposes (recreational, educational), periods (weekend, weekday), or combinations of these attributes. However, these inconsistencies did not demonstrate any clear pattern across studies. Studies applied various ways to examine the does effect of screen time, such as cumulative sums and ordinal or binary categories with different cutoffs. Lower ends of scree-time cutoffs in between-group comparisons that showed raised health risks are shown in Table 2.

### Pooled effect sizes

**Fig. 2:**
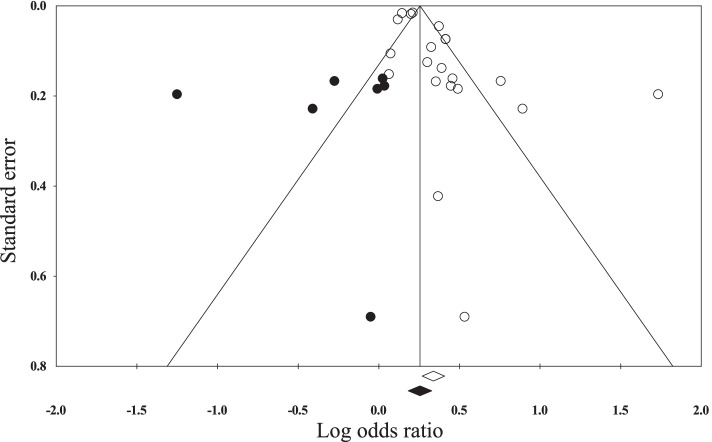
Funnel plot of standard error by log odds ratio. The while and dark circles represent observed studies and imputed counterparts, respectively. The white and dark diamonds are the overall effect size and the trimmed and filled effect size generated from random effects models, respectively

Three case-control and 16 cross-sectional studies provided 21 unique and valid adjusted odds ratios (ORs) of health risk comparisons using the screen time cutoff of 2 h per day. Not all health issues were included in the meta-analyses, because there were less than two studies with valid ORs per health issue. Within-study heterogeneity statistics were Q = 141.59, df (Q) = 20, *P* < 0.001, *I*^2^ = 85.9%, and tau^2^ = 0.013. The overall pooled adjusted odds ratio using the random effects model was 1.40 (95% CI = 1.31 to 1.50). Sensitivity analysis using the “one-study removed” approach resulted ORs ranged from 1.33 to 1.45, all *p* < 0.0001. The funnel plot was asymmetric (Fig. 2). The trim and fill analysis resulted an adjusted effect size of 1.29 (95% CI: 1.20, 1.39) after trimming 7 studies. The classic Fail-Safe N analysis showed that 2276 studies with a mean effect of zero would bring the overall effect to be statistically insignificant. The pooled effect sizes by health issues using fixed and random effects models are shown in Table 4. The overall pooled adjusted odds ratio based on the mixed effects analysis was 1.26 (95% CI = 1.21 to 1.31). The between-group heterogeneity is significant (Q = 25.8, df = 4, *P* < 0.001).

**Table 4:** Pooled effect sizes of overall studies and by health categories from fixed, random, and mixed effect models and heterogeneity statistics

Health categories	Study name	Odds ratio	95% CI	Weight (%)		Heterogeneity statistics			
			Lower	Fixed	Random	Q	df (Q)	*p*	*I* ^2^
Adiposity	Cheng, 2016 [35]	2.45	(1.56, 3.84)	7.7	14.6				
	Lin, 2018 [36]	1.59	(1.15, 2.18)	15.4	15.7				
	Wu, 2019 [37]	1.08	(0.88, 1.33)	35.5	16.5				
	Wu, 2018 [38]	1.07	(0.79, 1.44)	17.4	15.9				
	Lin, 2019 [39]	5.68	(3.85, 8.37)	10.4	15.2				
	Song Q, 2019 [40]	1.71	(0.44, 6.63)	0.9	6.6				
	Huang, 2013 [41]	1.57	(1.11, 2.23)	12.7	15.5				
	Fixed effect	1.53	(1.35, 1.73)			64.28	6	< 0.001	90.7
	Random effect	1.81	(1.16, 2.83)						
Cardio-metabolic risks	Wang M, 2019 [42]	1.45	(0.63, 3.33)	5.7	5.7				
	Li, 2017 [43]	1.36	(1.06, 1.74)	64.4	64.4				
	Wang, 2016 [44]	1.64	(1.14, 2.36)	29.9	29.9				
	Fixed effect	1.44	(1.18, 1.76)			0.71	2	0.701	0.0
	Random effect	1.44	(1.18, 1.76)						
Emotional problems	Qian, 2012 [45]	1.52	(1.31, 1.76)	35.0	35.0				
	Wang J, 2019 [46]	1.43	(1.03, 1.99)	6.9	6.9				
	Song Y, 2019 [47]	1.39	(1.16, 1.67)	23.0	23.0				
	Cao, 2011 [48]	1.52	(1.31, 1.76)	35.0	35.0				
	Fixed effect	1.48	(1.36, 1.62)			0.74	3	0.863	0.0
	Random effect	1.48	(1.36, 1.62)						
Myopia	Hu, 2020 [49]	1.46	(1.33, 1.60)	92.9	58.9				
	Pang, 2009 [50]	2.14	(1.54, 2.98)	7.1	41.1				
	Fixed effect	1.50	(1.37, 1.64)			4.83	1	0.028	79.3
	Random effect	1.71	(1.18, 2.47)						
Poor physical fitness	Huang, 2016 [51]	1.48	(1.13, 1.95)	0.5	2.2				
	Zhang, 2013 [52]	1.23	(1.18, 1.27)	24.7	25.4				
	Tian, 2017, aged 11–12 [53]	1.13	(1.06, 1.20)	9.4	18.9				
	Tian, 2017, aged 13–15 [53]	1.24	(1.20, 1.28)	34.9	27.1				
	Tian, 2017, aged 16–18 [53]	1.16	(1.12, 1.20)	30.5	26.5				
	Fixed effect	1.20	(1.18, 1.23)			14.76	4	0.005	72.9
	Random effect	1.20	(1.15, 1.25)						
Overall	Fixed effect	1.23	(1.21, 1.25)			141.59	20	< 0.001	85.9
	Random effect	1.40	(1.31, 1.50)						
	Mixed effect	1.26	(1.21, 1.31)			25.75	4	< 0.001	

## Discussion

### Main findings

The primary purpose of this review was to summarize the scope of health issues that have been tied with screen time and evidence of relationships from studies conducted among Chinese children aged 6–18 years. The literature retrieval returned a large volume of research which were not captured in previous reviews. For example, the so-far most comprehensive systematic review on the relationships between sedentary behavior and health indicators in school-aged children and youth only included 15 articles from mainland China and Taiwan [10], which is considerably fewer than the 252 articles retained in the present review. As a relatively exhaustive summary of Chinese studies on the topic of screen time and child health, the current review identified a similar collection of health issues and provided extra supportive evidence on health risks of excessive screen time [10, 30, 54, 55]. The meta-analyses also generated significant pooled effects of having screen time over 2 h per day on adiposity, cardio-metabolic risks, emotional problems, myopia, and poor physical fitness.

### Comparison with existing literature

Health impact of screen time on adiposity has received the most attention for Chinese school-aged children and adolescents, as a third of the included studies investigated relationships between screen time on adiposity. However, the proliferation of research in numbers did not improve the strength of evidence. Merely half of included studies demonstrated positive relationships between screen time and adiposity, while more than a dozen found no evidence. In addition, within-study inconsistencies varied across studies without clear patterns in terms of sample characteristics, screen types, and days of a week. Nevertheless, the pooled odds ratio of seven cross-sectional studies suggested significantly increased risks in overweight and/or obesity by the screen time cutoff of 2 h per day, which is comparable to the raised adiposity risks generated from 16 studies involving screen time 2 h per day (OR = 1.67, 95% CI = 1.48 to 1.88) and from 24 studies comparing the highest vs. lowest TV time per day (OR = 1.47, 95% CI = 1.33 to 1.62) without overlapping studies [20, 56]. The qualitative and quantitative results demonstrated small and somewhat inconsistent associations between screen time and adiposity, which corresponds with the conclusion made from a systematic review of 29 reviews [57].

Compared to adiposity, much fewer studies (89 vs. 10) focused on cardiometabolic disease risks. These studies showed relatively consistent associations between high levels of screen time and various cardiometabolic risk indicators, but some studies found that the associations became no longer significant after adjusting for BMI. A collective review of four systematic reviews with more cohort and longitudinal studies concluded weak evidence on associations between screen time and cardiometabolic disease risks [8]. In the present review, the pooled effect size from three studies examining the screen-time cutoff of 2 h per day showed a 44% increase in cardiometabolic risks (left ventricular hypertrophy, dyslipidemia, and non-alcoholic fatty liver disease), whereas a meta-analysis of odds ratios from 6 cross-sectional studies found no significant association with metabolic syndrome among 10–19 years old youth (OR = 1.20, 95% CI = 0.91 to 1.59) [58]. In terms of physical fitness, inverse associations with screen time were observed in all eight included studies, and the pooled effect size from three studies showed a 20% increase in poor physical fitness when screen time exceeded 2 h per day. Despite of the weak evidence strength, positive relationships identified for all three types of health issues indicate that screen time is likely to influence children’s weight status, cardiometabolic health, and physical fitness, possibly by displacing time spent on physical activity and sleep, and exposure to circumstances that facilitate consumption of less healthful food and beverages [59].

The etiology of myopia is different from aforementioned health issues. Besides genetics, time spent outdoors and on near work are strong causal factors associated with the onset of myopia [60], which involves a light-dependent dopaminergic mechanism or hyperopic defocus-induced eye growth [61, 62]. Excessive screen time can interact with both causal factors of myopia by competing with outdoor time and increasing near visual activity. However, a systematic review of 6 cohort studies and 9 cross-sectional studies found mixed associations between screen time and myopia and no association (OR = 1.02, 95% CI = 0.96 to 1.08) of the pooled effect from 1 cohort and 4 cross-sectional studies [22]. A pooled prevalence indicates that about 85% of Chinese adolescents would develop myopia at the high school-graduating age [16]. The potential impact of screen time on visual acuity has received great attention for Chinese children and adolescents. The number of myopia studies is second to adiposity in the present review and is much more than those identified in Lanca’s review (76 vs. 15) which has only one overlapping study [63]. The mixed evidence is likewise to Lanca’s review as the majority of studies showed positive relationships while null and inconsistent findings were found in all three cohort studies and a number of cross-sectional studies. Moreover, an inverse association were found in one study with a possible explanation that parents of myopes were likely to restrict their children’s screen time due to the fear of myopia progression [64]. The reciprocal relationship between myopia diagnosis and management that involving restricting screen time challenges the investigation of causal relationships. Current level of evidence needs to be strengthened with more robust findings from cohort and experimental studies.

Existing evidence from previous reviews of primarily cross-sectional studies points out that levels of evidence on positive associations between screen time and psycho-behavioral issues were from moderate to strong in depressive symptoms [55, 65], health-related quality of life [66, 67], poor psychological wellbeing [55, 66], and hyperactivity and inattention [10, 66, 68], from low to moderate in self-esteem [10, 30, 65, 66], and insufficient in other mental and behavioral problems of self-injury, suicidal ideation, and eating disorders [65, 66]. In contrast, a more recent review of longitudinal studies found that the prospective relationships between screen time and depression were weak and varied by screen types, and relevant evidence was insufficient on anxiety or self-esteem [26]. In the present review, studies examined the relationship of screen time with various psycho-behavioral issues. One quasi-experimental study and two cohort studies provided some evidence on the temporal relationships between screen time and scores of Symptom Checklist-90, internet addition, and depressive symptoms within time spans of three months, 12 months, and five years, respectively [69–71]. The rest of cross-sectional studies predominately showed positive relationships of all sorts of screen time with depressive symptoms, anxiety, mental wellbeing, conduct problems, attention deficiency, but found no or inconsistent associations between screen time and dyslexia, executive function, self-injury, and adaptability.

Sleep and physical activity may ameliorate the adverse relationships between screen time and mental health problems. Isotemporal substitution modeling of cross-sectional data from Canadian adolescents showed that replacing screen time with sleep and physical activity in a short interval of 15 min would associate with better mental health outcomes of anxiety, depression, and functioning [25]. Consistent with previous reviews [55, 72], we found positive evidence on the relationship between screen time and sleep disorders among Chinese children and adolescents based on nine cross-sectional observations that all except one only examined TV viewing time. Intervention studies showed promising results in increasing sleep duration through screen time reduction [23]. The interlocking relationships between screen time and sleep provide empirical implications on behavior-associated health issues.

A few studies examined the potential impact of screen use on musculoskeletal health among Chinese adolescents, and all demonstrated positive associations of time spent on TV, computer, and other hand-held devices with some kinds of musculoskeletal injuries such as neck and shoulder discomforts, wrists and hand pain, and lower back pain. Two main drawbacks of these studies include the reliance on participants’ self-reports of musculoskeletal discomforts and the cross-sectional study design. An earlier review of physical examination-diagnosed musculoskeletal disorders have demonstrated some evidence on associations between computer work and musculoskeletal discomforts in adults [73]. In addition to total time spent on screen-based devices, studies suggest that the potential influence of screen time on musculoskeletal health vary by device types, postures, tasks, and durations and frequencies of device usage [74]. Besides, there still lacks evidence on identifying screen time as a prognostic factor of musculoskeletal pain among the young [21].

The present review found a relatively consistent inverse relationship between screen time and school exam scores in 2 longitudinal and 6 cross sectional studies, except one study showing a positive prospective relationship between weekend TV viewing time and math exam scores among students living in rural northwestern China [75]. Two previous reviews identified 41 studies outside China [10, 30]. Among these studies, four in the longitudinal design found that longer TV viewing time was associated with attention difficulties or lower academic achievement, but less consistent findings were found in cross-sectional studies and with other screen types. In the present review, most studies of academic achievement investigated total screen time, thus the results cannot generalize to specific screen types. In contrast, another subset of studies primarily focused on internet surfing time and demonstrated its negative relationship with sub-health which was assessed by a self-reported questionnaire of the 3-month presence of 71 sub-optimal physical and mental conditions [76]. In addition, a scattering number of studies showed interests in time spent on various types of screen-based activities, such as TV viewing, internet surfing, electronic gaming, and their relationships with physical or pubertal growth, injury, and respiratory health. These health issues are not included in previous reviews [8], and the mechanisms related to which are unclear.

### Strengths and limitations

Although the protocol was not registered in the PROSPERO database, this review was conducted following rigorous methodological procedures [9, 10]. Peer-reviewed articles written in Chinese and English were retrieved from five major electronic databases in a relatively comprehensive fashion without imposing initial publication dates and specific health categories. However, more studies from Hong Kong, Macau, and Taiwan would be retained if electronic databases of traditional Chinese literatures were included. Each step of article selection, information extraction, and study quality appraisal involved collaborations among two to three reviewers to ensure data quality. Unfortunately, the level of evidence was limited by the design of included studies, as 89.5% of which were cross-sectional. The nature of the primary research design prevents any inference on causality in the relationships between screen time and health issues. Moreover, only 8.6% of included studies were classified as high research grades, which compromised the quality of evidence. One of the common issues that down-grading research quality was the reliance on self-reported measures of screen time without reporting psychometric properties of the instruments. The other was without adequate considerations of confounders in the examination of relationships between screen time and health issues.

In addition, we observed vast and various heterogeneities in the inclusion of screen-based activities, analytical treatment of screen time, and reporting of study findings, which prevents across-study comparisons. Even though we were strict about including effect sizes of odds ratios generating from the screen-time (combinations of TV/video viewing time and at least another type of non-TV screen time) cutoff of 2 h per day in the quantitative synthesis, the fixed and mixed effect models demonstrated significant between-study and between-group heterogeneities, respectively. The funnel plot was also not symmetric; thus, we performed the trim and fill analysis to adjust for the potential publication bias. Moreover, we purposefully focused on studies conducted among Chinese children and adolescents so that the results cannot be generalized to other populations.

## Conclusions

In summary, findings from Chinese children and adolescents are generally congruent with those from the English literature, which connects screen time with adverse physical, mental, and behavioral health conditions. This review made a modest contribution to the evidence base of the potential health impact of screen time due to the additions of both positive and inconsistent findings from primarily cross-sectional studies. Beyond findings on cross-sectional relationships, future studies need to investigate the potential dose effect and mechanisms of screen time on specific health issues so that more evidence-based practical recommendations other than no more than two hours of daily screen time can be made.

## Supplementary Information

**Additional file 1: Table A.1. **Search strategies.** Table A.2.** Data extraction table.

## Data Availability

Supplementary data to this article can be found online. Additional data will be available upon requests to Youjie Zhang (ujzhang@suda.edu.cn).

## Abbreviations

CNKI: China National Knowledge Infrastructure
OR: odds ratio
CI: confidence interval
C: computer
M: mobile phone
I: internet
G: electronic games
TV: television
ST: screen time
non-TV ST: screen time excluding TV watching
