# Supplementary material for: Screen time and health issues in Chinese school-aged children and adolescents: a systematic review and meta-analysis
Source: BMC Public Health. 2022 Apr 22;22:810. doi: 10.1186/s12889-022-13155-3 (PMC9034635; doi:10.1186/s12889-022-13155-3)
Supplement: Supplementary file 1 — Additional file 1: Table A.1. Search strategies. Table A.2. Data extraction table. [file 12889_2022_13155_MOESM1_ESM.docx]

Table A.1 Search strategies

| Databases | Search terms |
| --- | --- |
| China National Knowledge Infrastructure | 第一步 限定人群  SU = ('儿童' + '青少年' +'小学生'+ '中学生' + '中小学生' + '初中生' + '高中生' - '学龄前') OR TI = ('儿童' + '青少年' +'小学生'+ '中学生' + '中小学生' + '初中生' + '高中生' - '学龄前') OR KY = ('儿童' + '青少年' +'小学生'+ '中学生' + '中小学生' + '初中生' + '高中生' - '学龄前')  第二步 筛选行为（在第一步结果中检索）  SU = ('视屏' + '静态' + '屏幕' + '静坐' + '久坐' + '网络' + '上网' + '电视' + '手机' + '平板' + '电脑' + '电子产品') OR AB = ('视屏' + '静态' + '屏幕' + '静坐' + '久坐' + '网络' + '上网' + '电视' + '手机' + '平板' + '电脑' + '电子产品')  第三部 限定摘要关键词汇 （在第二步结果中检索）  AB = ('时间' + '时长' + '小时' + '分钟' + 'hours' + 'hour' + 'hr' + 'minutes' + 'min' + 'minute')  English translation of above search strategies is shown below. Please note that Chinese literature retrieval should only be replicated using Chinese search terms.  First step: limit study population  SU = ('children' + 'adolescents' +' pupils'+ 'middle school students' + 'primary and secondary school students' + 'junior high school students' + 'senior high school students' - 'preschool ') OR TI = ('children' + 'adolescents' +'pupils'+ 'middle school students' + 'primary and secondary school students' + 'junior high school students' + 'senior high school students' - 'preschool')  Second step: refine behaviors from search results of step one  SU = ('screen' + 'sedentary' + 'digital screen'+' sit '+' prolonged sitting '+' internet '+' online '+' TV '+' mobile phones' + 'tablet' + 'computer' + 'electronics') OR AB =('screen' + 'sedentary' + 'digital screen'+' sit '+' prolonged sitting '+' internet '+' online '+' TV '+' mobile phones' + 'tablet' + 'computer' + 'electronics')  Third step: limit keywords in abstracts from search results of step two  AB = ('time' + 'length of time' + 'hours (in Chinese)' + 'minutes (in Chinese)' + 'hours' + 'hour' + 'hr' + 'minutes' + 'min' + 'minute') |
| Wanfang | (主题:( "儿童" + "青少年" +"小学生"+ "中学生" + "中小学生" + "初中生" + "高中生")^"学龄前"+题名或关键词: ( "儿童" + "青少年" +"小学生"+ "中学生" + "中小学生" + "初中生" + "高中生")^"学龄前")*(主题:("视屏" + "静态" + "屏幕" + "静坐" + "久坐" + "网络" + "上网" + "电视" + "手机" + "平板" + "电脑" + "电子产品")+摘要:("视屏" + "静态" + "屏幕" + "静坐" + "久坐" + "网络" + "上网" + "电视" + "手机" + "平板" + "电脑" + "电子产品"))*摘要:("时间" + "时长" + "小时" + "分钟" + "hours" + "hour" + "hr"+ "minutes" + "min" + "minute")  限定至中文文章  English translation of above search strategies is shown below. Please note that Chinese literature retrieval should only be replicated using search strategies in Chinese.  (topic: (" children "+" adolescents "+" pupils"+" middle school students "+"primary and secondary school students "+" junior high school students "+"senior high school students") ^ "preschool" + title or keywords: (" children "+" adolescents "+" pupils"+" middle school students "+"primary and secondary school students "+" junior high school students "+"senior high school students") ^ "preschool" * (topic: (" screen "+" sedentary ", "digital screen" + "sit", "prolonged sitting" + "network", "Internet" + "television" + "mobile phone" + "tablet" + "computer" + "electronics") + abstract: (" screen "+" sedentary ", "digital screen" + "sit", "prolonged sitting" + "network", "Internet" + "television" + "mobile phone" + "tablet" + "computer" + "electronics")) * abstract: ("time" + "length" + "hour (in Chinese)" + "minutes (in Chinese)" + " hr "+" minutes "+" min ", "minute")  Limit to articles written in Chinese |
| PubMed | Search: ("sedentaries"[Title/Abstract] OR "sedentariness"[Title/Abstract] OR "sedentary"[Title/Abstract] OR"sitting position"[MeSH Terms] OR "sitting"[Title/Abstract] OR "position"[Title/Abstract] OR "sitting position"[Title/Abstract] OR "sitting"[Title/Abstract] OR "sittings"[Title/Abstract] OR "inactiv*"[Title/Abstract] OR "media"[Title/Abstract] OR "media s"[Title/Abstract] OR "medias"[Title/Abstract] OR "screen time"[Title/Abstract] OR "screen-based"[Title/Abstract] OR "screen-based"[Title/Abstract] OR "computer*"[Title/Abstract] OR "video*"[Title/Abstract] OR "tablet*"[Title/Abstract] OR "smartphone*"[Title/Abstract] OR "smart phone*"[Title/Abstract] OR "mobile phone*"[Title/Abstract] OR "mobile phone*"[Title/Abstract] OR "cellphone*"[Title/Abstract] OR "cell phone*"[Title/Abstract] OR "television*"[Title/Abstract] OR "tv"[Title/Abstract] OR "low energy expenditure"[Title/Abstract] OR "electronic gam*"[Title/Abstract] OR "internet"[Title/Abstract]) AND ("China"[MeSH Terms] OR "China"[Title/Abstract] OR "China"[Title/Abstract] OR "China"[Title/Abstract] OR "Chinese"[Title/Abstract] OR "Chinese"[Title/Abstract] OR "Hong Kong"[MeSH Terms] OR "Hong Kong"[Title/Abstract] OR "Macaw"[Title/Abstract] OR "Macaws"[Title/Abstract] OR "Taiwan*"[Title/Abstract]) AND ("adolescent*"[Title/Abstract] OR "child*"[Title/Abstract] OR "adolescent"[MeSH Terms] OR "adolescent"[Title/Abstract] OR "youth"[Title/Abstract] OR "youths"[Title/Abstract] OR "youth s"[Title/Abstract] OR "student*"[Title/Abstract]) Filters: Journal Article, Humans, English, Child: 6-12 years, Adolescent: 13-18 years |
| Web of Science | You searched for: (TS=(“sitting position” OR sedentar* OR sitting* OR inactiv* OR media OR “screen time” OR "screen-time" OR "screen based” OR screen-based OR computer* OR video* OR tablet* OR smartphone* OR “smart phone*” OR mobile phone* OR “mobile phone*” OR cellphone* OR “cell phone*” OR television* OR tv OR “low energy expenditure” OR “electronic gam*” OR internet) AND TS=(China* OR Chinese* OR “Hong Kong” OR Macaw OR Taiwan*) AND TS=(child* OR adolescent* OR youth* OR student*)) AND LANGUAGE: (English) AND DOCUMENT TYPES: ( ARTICLE OR PROCEEDINGS PAPER OR EARLY ACCESS )  Timespan: All years. Indexes: SCI-EXPANDED, SSCI, CCR-EXPANDED, IC. |
| Embase | 1 sedentar*.tw.  2 sitting.tw.  3 inactiv*.tw.  4 media.tw.  5 screen-time.tw.  6 screen-based.tw.  7 "screen time".tw.  8 "screen based".tw.  9 computer*.tw.  10 video*.tw.  11 tablet*.tw.  12 smartphone*.tw.  13 "smart phone*".tw.  14 cellphone*.tw.  15 "cell phone*".tw.  16 mobile phone*.tw.  17 "mobile phone*".tw.  18 television*.tw.  19 tv.tw.  20 "low energy expenditure".tw.  21 1 or 2 or 3 or 4 or 5 or 6 or 7 or 8 or 9 or 10 or 11 or 12 or 13 or 14 or 15 or 16 or 17 or 18 or 19 or 20  22 China*.tw.  23 Chinese*.tw.  24 "Hong Kong".tw.  25 Macaw*.tw.  26 Taiwan*.tw.  27 22 or 23 or 24 or 25 or 26  28 child*.tw.  29 adolescen*.tw.  30 youth*.tw.  31 student*.tw.  32 28 or 29 or 30 or 31  33 21 and 27 and 32  34 limit 33 to (human and English language and embase and (article or article in press) and journal and (school child <7 to 12 years> or adolescent <13 to 17 years>)) |

Table A.2 Data extraction table

| **Article no.** | **First author** | **Year** | **Language written** | **Study design** | **Location** | **Age range** | **sample size** | **Health issues** | **Health indicators** | **Types of screen time** | **Adjusted covariates** | **Main results** | **Lower end of screen time cutoffs** | **Quality score** | **Quality rating** |
| --- | --- | --- | --- | --- | --- | --- | --- | --- | --- | --- | --- | --- | --- | --- | --- |
| 1 | Jia Li | 2017 | Chinese | Cross-sectional | Beijing | 4-16 years | 2826 | 2-myopia | Axial length, corneal curvature | TV; eletronic devices | gender, paternal myopia, etc. | Conrneal curvature was inversely associated with TV viewing time (b=-0.102，95% CI：-0.141～-0.064) . Axial length was not associated with TV viewing time.  Conrneal curvature and Axial length was not associated with non-TV screen time. | none | 13 | moderate |
| 2 | Fangru Yang | 2005 | Chinese | Intervention | Hunan Changsha | 12-19 years | 52 | 3-psychobehavioral problems | mental health | Internet | none | After 3-month intervention, hours and frequency of internet surfing decreased significantly (t=9.39, 6.45; P<0.01), SCL-90 scores decreased significantly (169.34±44.02 vs. 138.30±36.13, p< 0.01). | none | 8 | low |
| 3 | Yuchan Huang | 2018 | Chinese | Case-control | Hubei | 1-12 years | 2114 | 2-myopia | Myopia | TV | none | Myopia rates between TV >=1 vs.<1h/d: 73.25% vs.26.75%, x^2 = 81.60, p<0.05. | none | 11 | low |
| 4 | Yuanyuan Zhang | 2018 | Chinese | Cross-sectional | Zhejiang Ningbo | Grades 7-12 | 3264 | 3-psychobehavioral problems | depression | cellphone | parental education, grade level, gender | depression was positively associated with cellphone use (b=0.184, p <0.001). | none | 10 | low |
| 5 | Yuanyuan Zhang | 2018 | Chinese | Cross-sectional | Zhejiang Ningbo | Grades 7-12 | 3402 | 3-psychobehavioral problems | anxiety | cellphone | parental education, gender, academic pressure, academic interests, boarding school | Anxiety was positively associated with cellphone use (b=0.12, p <0.001) | none | 12 | moderate |
| 6 | Xiaomei Lei | 2008 | Chinese | Cross-sectional | Shangxi Xi'an | Grades 1-6 | 2037 | 6-Sleep disorders | sleep disorders | TV | parental education, homework load, family bedtime | TV viewing hours was positively associated with sleep disorder (OR=2.96, p < 0.05). | none | 7 | low |
| 7 | Lingli Xu | 2012 | Chinese | Intervention | Guangzhou | n.a. | 177 | 1-adiposity | overweight and obesity | TV, computer, cellphone, intervent, gaming, etc. | none | Pre- and post-intervention analysis showed a significantly decrease in daily screen time (P < 0.01) . Rates of overweight and obesity were 13.33% and 6.25% at pre-test and 10.00% and 4.69% at post-test, p > 0.05. | none | 11 | low |
| 8 | Ying Zhao | 2019 | Chinese | Cross-sectional | An Hui | Grades 7-12 | 3477 | 9-sub health | Physical and mental sub-health | TV, Cellphone, MP4, DVD/VCD | school grade, parental education, family income, number of friends, parental expectation, study burden, and academic performance | Weekday screen time >2h/d vs. <= 2h/d OR(95%CI): 1.25 (1.02～1.53) for physical sub-health and 1.48 (1.19～1.82) for mental sub-health. Weekend day screen time >2h/d vs. <= 2h/d OR(95%CI):1.44(1.22～1.69) for physical sub-health and 1.72(1.44～2.05) for mental sub-health. Weekday and weekend screen time both >2h/d, one >2h.d vs. both <= 2h/d OR(95%CI): 1.63(1.29~2.70)and 1.34 (1.12~1.60) for physical sub-health, 2.13(1.65~2.74) and 1.64(1.35~1.99) for mental sub-health. | ST <=2h/d; weekday and weekend ST ST <= 2h/d | 12 | moderate |
| 9 | Lijing Wu | 2015 | Chinese | Cross-sectional | Beijing | Grades 3-5 | 1472 | 3-psychobehavioral problems | depression | TV/videos, computer/e-games/iPad | school, age | Screen time (unknown coding): depression OR (95%CI) were 2.48 (1.69～3.64) in boys and 3.04 (1.80～5.12) in girls. | none | 12 | moderate |
| 10 | Yi Song | 2012 | Chinese | Cross-sectional | 30 provinces | 9-18 years | 149611 | 1-adiposity | overweight and obesity | TV, e-games, computer | age, urban, provinc, gender, ageetc.. | Screen time >2 vs. <=2h/d: overweight and obesity OR (95%CI) 0.91 (0.85～0.97) in boys , insignificant in girls.  Screen time >2 vs. <=2h/d: overweight and obesity OR (95%CI) 0.86 (0.7～0.99) in rural students，insignificant in urban students. Screen time >2 vs. <=2h/d: overweight and obesity OR (95%CI) 0.82 (0.69～0.97) in 13-15 years old students，insignificant in 9-12 years old and 16-18 years old students. | screen time <=2h/d | 12 | moderate |
| 11 | Zhanzhong Cao | 2008 | Chinese | Cross-sectional | Ningxia | Grades 7-12 | 505 | 2-myopia | Low vision | TV, e-games | none | Myopia rates between ST >=0.5 vs. < 0.5h/d: p > 0.05 | none | 4 | low |
| 12 | Hong Liu | 2015 | Chinese | Cross-sectional | Yunan | 7-18 years | 7200 | 1-adiposity | overweight and obesity | electronic devices | gender, age, urban, daily physical activity hours, sleep. | TV <2 vs. >=2h/d：overweight OR (95%CI) was 1.107 (0.861～1.424) , obesity OR (95%CI) was 0.796 (0.567～1.118) . Non-TV screen time <2 vs. >=2h/d: overweight OR (95%CI) was 0.960 (0.768～1.4201); obesity OR (95%CI) was 0.756 (0.554～1.031) . | TV < 2h/d; non-TV screen time < 2h/d | 13 | moderate |
| 13 | Budan Hu | 2017 | Chinese | Cross-sectional | Szechwan | Grades 7-9 | 4818 | 2-myopia | Low vision | computer, TV | none | Myopia rates between TV >=15h/wk vs. <15h/wk: 41.23% vs. 38.38%, x^2 = 4.003，P=0.045. | none | 13 | moderate |
| 14 | Feng Long | 2018 | Chinese | Cross-sectional | Beijing | 17-20 years | 5708 | 8-musculoskeletal injuries | spinal pain | TV, computer ipad and cellphone | backpack weight multiple carrying time, physical activity, sitting posture time. | accumulative using time of electronic devices (3296，8108，8108 vs. 1070 hours): spinal pain OR(95%CI) were 1.308(1.006～1.701), 1.350(1.021～1.786), 1.474(1.114～1.950). | accumulative screen time <=1070 hours | 11 | moderate |
| 15 | Yuntao Wang | 2019 | Chinese | Cross-sectional | Macau | 7-22 years | 4473 | 1-adiposity | BMI | TV, video, computer games | sleep hours, outdoor time, weekly physical activity frequency and time, homework hours, breakfast frequency, frequency of meal away from home | Daily screen time was positively associated with BMI (b=0.159, P<0.01) . | none | 11 | moderate |
| 16 | Yanqing Zhang | 2016 | Chinese | Cross-sectional | Shandong Zibo | 10-15 years | 700 | 2-myopia | Low vision | cellphone, TV | none | Myopia rates between cellphone＜1h, 1-2h, >2 h/d: 23.10%, 67.54%, 72.32%，x^2 = 121.41, P<0.01;  Myopia rates between TV＜1h, 1-2h, >2 h/d: 50.77%， 65.37%， 81.17%，x^2 = 39.01，P<0.01. | none | 8 | low |
| 17 | Fei Wang | 2019 | Chinese | Cross-sectional | Hunan | 8-20 years | 8129 | 2-myopia | Low vision | Internet | outdoor time, caregivers' reminder of postures，homework hours | Internet >=2h vs.<2h/d: myopia OR (95%CI) was 1.358 (1.224～1.507) . | Internet <2h/d | 12 | moderate |
| 18 | Shuo Wang | 2020 | Chinese | Cross-sectional | Beijing | Grade 7 | 1405 | 2-myopia | Myopia | cellphone or other eletronic devices; TV | none | Non-TV sceen time between myopia and non-myopia group : 4.0±4.50 vs. 4.0±4.50, Z=-0.804, P=0.421) . TV viewing hours between myopia and non-myopia group : 2.0±4.50 vs. 2.67±4.50, Z=-3.488,P<0.001) .  Multiple logistical regression: null. | none | 12 | moderate |
| 19 | Chong Xu | 2015 | Chinese | Case-control | Guangdong shenzhen | 4-12 years | 600 | 2-myopia | Refractive error | eletronic devices | none | sceen time between myopia and non-myopia group : 4.22±0.69 vs. 0.53±0.72 h/d, t = 23.6, p < 0.05. | none | 9 | low |
| 20 | Jianhui Huang | 2016 | Chinese | Cross-sectional | Beijing | 9-22 years | 7136 | 2-myopia | Low vision | TV; cellphone, tablet, e-games, e-readers, videos | sleep hours, breakfast intake, diary intake, PE class frequency, etc. | TV <0.5 h/d, 0.5-1 h/d vs. >=1h/d: myopia OR (95%CI) were 1.633 (1.433～1.859) , 1.371 (1.186～1.585) . non-TV ST<0.5 h/d, 0.5-1 h/d vs. >=1h/d: myopia OR (95%CI) were 0.731 (0.646～0.828) , 0.797 (0.702～0.906) . | TV <1h/d; non-TV screen time <1h/d | 13 | moderate |
| 21 | Qingwen Qian | 2012 | Chinese | Cross-sectional | Anhui | Grades 7-9 | 5268 | 1-adiposity | obesity | TV, internet | none | Weekday TV viewing >1 h/d in obese and non-obese group: 15.6% vs. 17.4%, χ2 = 1.02, p =0.312. Weekend TV viewing >2 h/d in obese and non-obese group: 27.9% vs. 23.9%, χ2 = 3.93, p = 0.047. Weekday Internet surfing >0.4 h/d in obese and non-obese group: 29.0% vs. 27.1%, χ2 = 0.72m, p = 0.396. Weekend Internet surfing >2h/d in obese and non-obese group: 39.2% vs. 31.4%, χ2 = 12.32, p = 0.001. | none | 9 | low |
| 21 | Qingwen Qian | 2012 | Chinese | Cross-sectional | Anhui Bengbu | Grades 7-9 | 5268 | 3-psychobehavioral problems | depression, anxiety, dissatisfaction toward school life | TV, Internet | gender, grade, family type, family income, obesity, fruit and vegetables intake, sugary drink intake. | Screen time >2h/d vs. <=2 h/d, depression, anxiety, dissatisfaction toward school life OR (95%CI) were1.52 (1.31～1.76), 1.36 (1.18～1.57), 2.07 (1.79～2.40). | screen time <=2h/d | 11 | moderate |
| 22 | Guangyu Hao | 2009 | Chinese | Cross-sectional | Inner mongolia | 6-14 years | 1275 | 2-myopia | Myopia | computer | age, reading light, reading posture, parental education, parental myopia, study desk condition. | Computer >1h/time vs. 1h/time: myopia OR (95%CI) was 3.782 (2.424～5.902) . Average computer time was not associated with myopia rates. | computer < 1h/time | 9 | low |
| 23 | Xuefeng Chai | 2015 | Chinese | Cross-sectional | Inner mongolia | 6-12 years | 4560 | 2-myopia | Myopia | TV | parental myopia, reading distance, homwork hours, outdoor time, picky eating | TV >=2h/d vs. <2 h/d: myopia OR (95%CI) was 1.851 (1.420～2.362) . | TV <2h/d | 11 | moderate |
| 24 | Xian Gao | 2016 | Chinese | Cross-sectional | Beijing | 7-12 years | 1370 | 1-adiposity | Obesity | TV, computer | none | TV > 1 h/d vs. <= 1h/d: obesity rates (χ2 = 0.487, p =0.485);  computer > 1 h/d vs. <= 1h/d: obesity rates(χ2 = 2.260, p =0.133) . | none | 10 | low |
| 25 | Zhiwei Xia | 2018 | Chinese | Cross-sectional | Beijing | 9-22 years | 20255 | 2-myopia | Low vision | eletronic devices | gender, location of residence, outdoor time, reading posture, reading light, homework hours, after-school classes, sleep hours | ST >=1h/d vs. <1h/d: myopia OR (95%CI) was 1.24 (1.09～1.41) in total sample. 1.15 (0.99 - 1.33) in 10-12-year-olds, 1.50 (1.09-2.07) in 13-15 years, 1.51 (1.14-1.88) in 16-18-year-olds. | screen time <1h/d | 12 | moderate |
| 26 | Jingxiong Jiang | 1999 | Chinese | Case-control | Beijing | 6-12 years | 411 | 1-adiposity | Obesity | TV | eating speed, snacks before supper, after-class physical activity, intake frequencies of sugar sweetened beverages, lunch at school, parents with obesity | TV viewing time >1.5h/d vs. <=1.5h/d, OR (95%CI) :1.76 (1.13～2.71) for obesity | TV <=1.5h/d | 12 | moderate |
| 27 | Yin Guo | 2012 | Chinese | Cross-sectional | Beijing | 5-13 years | 681 | 2-myopia | Myopia | TV, eletronic devices | age, parental myopia, outdoor time, outdoor time, study time. | TV (h/d): myopia OR (95%CI) was 0.66 (0.52～0.84) . ST (h/d) : myopia OR (95%CI) null. | none | 12 | moderate |
| 28 | Yali An | 2005 | Chinese | Cohort | Heilongjiang Daqing | Baiseline: 5-6 years, follow-up: 10-11 years. | 400 | 1-adiposity | Body weight | TV | Age, gender, baseline weight, genotype, family income | Body weight at 5-year follow-up was significantly positively associated with weekly TV viewing time. But BMI at 5-year follow-up was not associated with weekly TV viewing time. | none | 9 | low |
| 29 | Xian Gao | 2012 | Chinese | Cross-sectional | Beijing | 11-20 years | 1930 | 1-adiposity | overweight and obesity | TV or video, e-games including gaming consoles, cellphone and computer, internet | gender, school, maternal education, family structure, self-reported academic performance, physical activity | TV viewing time 2-3h/d, >4h/d vs. <1h/d: obesity OR (95%CI) were 0.893 (0.615～1.298) and 1.755 (1.035～2.976) . Gaming time 2-4h/d, >4h/d vs. <1h/d: obesity OR p > 0.05.  Internet 2-4h/d, >4h/d vs. <1h/d: obesity OR p > 0.05. | TV <1h/d, e-games <1h/d , internet <1h/d | 11 | moderate |
| 30 | Huiping Song | 2010 | Chinese | Cross-sectional | Beijing | Grades 1-12 | 15316 | 2-myopia | Myopia | TV, computer | school type, location of residence, age, parental education, reading distance, eading posture, 每天study hours, watching distance, parental myopia | Screen time (1= <1 h, 2= 1～2 h, 3= 2～3 h, 4= 3～4 h, 5= >=4 h, ordinal coding): myopia OR (95%CI) was 0.918 (0.879～0.958). | none | 11 | moderate |
| 31 | Xiaoli Pan | 2001 | Chinese | Cross-sectional | Szechwan | 9-13 years | 694 | 3-psychobehavioral problems | mental health | TV | school type, gender , paternal occupation, parental relationships, death of caregivers, leisture activity hours, psychiatric tendency, emtional stability | MHT scores were positively associated with TV viewing hours (beta=1.126, p< 0.001). | none | 6 | low |
| 32 | Jue Liu | 2017 | Chinese | Cross-sectional | Beijing | 7-11 years | 1574 | 1-adiposity | Obesity | TV/video, computer/games/Ipad | none | Studemts having screen time >2h/d vs. <=2h/d had greater overweight and obesity rate (42.94% vs. 30.94%, p < 0.01) and obesity rate (27.03% vs. 16.60%, p < 0.01) . | none | 12 | moderate |
| 33 | Meijing An | 2018 | Chinese | Cross-sectional | Beijing | 7-18 years | 2670 | 1-adiposity | overweight and obesity | TV, computer, electronic games | urbancity, gender, school, grade, non-screen sedentary time, low intensity physical activity, MVPA. | Screen time 2～3h/d, >3h/d vs. <=2h/d, OR (95%CI) : 1.388 (1.014～1.902) and 1.590 (1.037～2.437) for overweight and obesity. Only weekday screen time was significant, weekend screen time was not significant. | screen time <=2h/d | 13 | moderate |
| 34 | Yan Wu | 2020 | Chinese | Cross-sectional | Beijing | 9-22 years | 1450 | 2-myopia | Low vision | computer, tablets | gender, homework hours, sleep hours, parental myopia | Non-TV ST <1h, 1-2h vs. >=2h: myopia OR (95%CI) were 0.601 (0.391～0.924) , 0.619 (0.337～0.918) . Non-TV ST <1h, 1-2h vs. >=2h: myopia OR (95%CI) were 0.493 (0.370～0.656) , 0.561 (0.412～0.763) . | non-TV screen time <2h/d | 12 | moderate |
| 35 | Weiguo Lu | 2014 | Chinese | Cross-sectional | Fofan | 15-18 years | 7764 | 1-adiposity | BMI | Cellphone, computer | none | Screen time between BMI groups (<24, 24～<28, >=28) was not significantly different . | none | 9 | low |
| 36 | Yi Wang | 2014 | Chinese | Cross-sectional | Urumuqi | 6-13 years | 1611 | 1-adiposity | Obesity | TV | school type, age, gender, breakfast location, weekend homework load, etc. | TV time was not associated with obesity rates. | none | 13 | moderate |
| 37 | Juanyuan Yang | 2014 | Chinese | Cross-sectional | Beijing | Grades 3-6 | 722 | 2-myopia | Low vision | computer | gender, parental myopia, reading distance, physocal activity | Computer 0.5-1h/d, 1-2h/d, >=2h/d vs. <0.5h/d: myopia OR (95%CI) : 2.23 (1.36～3.65) , 1.62 (0.99～2.65) , 1.44 (0.84～2.46) . | computer <0.5h/d | 11 | moderate |
| 38 | Ping Shi | 2013 | Chinese | Cross-sectional | Beijing | Grades 7-12 | 1232 | 1-adiposity | overweight and obesity | TV, video, computer, e-games | gender, sweets intake, fried food intake, street food intake, physical activity | Computer >2h/d, TV >2h/d, e-games >2h/d : overweight and obesity OR (95%CI) were 4.301 (3.077～6.014) , 3.877 (2.839～5.295) , 6.552 (2.538～16.910) , all p <0.001. | Computer <=2h/d; TV <=2h/d; e-games <=2h/d | 14 | moderate |
| 40 | Liang Ma | 2014 | Chinese | Cross-sectional | Beijing | 11-18 years | 2261 | 1-adiposity | overweight and obesity | TV, computer | gender, parental obesity, physical activity habits | TV viewing time <1h/d, 1h/d, 2h/d vs. >2h/d: overweight OR (95%CI) were 0.152 (0.08～0.31) , 0.796 (0.55～1.15) , 0.322 (0.19～0.54) ; obesity OR (95%CI) were 0.197 (0.12～0.34) , 0.239 (0.16～0.36) , 0.424 (0.28～0.64) . Computer using time <1h/d, 1h/d, 2h/d vs. >2h/d: overweight OR p > 0.05, obesity OR (95%CI) were 0.256 (0.15～0.44) , 0.201 (0.13～0.31) , 0.647 (0.43～0.97) . | TV <=1h/d; Computer <=2h/d | 14 | moderate |
| 41 | Chenchen Ceng | 2018 | Chinese | Cross-sectional | Anhui Bengbu | Grades 1-2 | 1269 | 3-psychobehavioral problems | executive function | TV , computer, tablet | age, gender, parental education, family income, BMI | weekday screen time (>=2, 1-2, 0.5-1, 0-0.5 vs. none) was not associated with executive function scores (p > 0.05).  weekend screen time (>=2, 1-2, 0.5-1, 0-0.5 vs. none) was not associated with executive function scores (p > 0.05). | weekday and weekend screen time = 0 | 11 | moderate |
| 42 | Wenli Liu | 2017 | Chinese | Cross-sectional | Hunan Changsha, Xiangtan | 11-20 years | 1034 | 1-adiposity | overweight and obesity | TV, e-games, internet | none | Screen time was not different by weight status. | none | 7 | low |
| 43 | Hongmei Xue | 2014 | Chinese | Cross-sectional | Chengdu | 7-15 years | 2211 | 1-adiposity | overweight and obesity | TV; Computer | none | weekday TV(computer) <2 vs. >=2h/d : overweight and obesity rate (p > 0.05) in 7-15 years old boys and girls.  weekend TV(computer) <2 vs> =2h/d: overweight and obesity rate (p > 0.05) in 7-15 years old boys and girls. | none | 10 | low |
| 44 | Dan Tang | 2018 | Chinese | Cross-sectional | Chengdu | 6-12 years | 2658 | 10-Miscellaneous | Height | online games and TV | none | Significant group difference in higher than genetic height were found by TV viewing time (<10, 10-20, >20 h/week): 70.7%, 26.5% vs. 2.8%, χ2 = 6.78，P＜0.001. | none | 10 | low |
| 47 | You Li | 2013 | Chinese | Cross-sectional | Dali | 7-13 years | 4354 | 1-adiposity | overweight and obesity | TV | gender, age, family income, picky eating, snack intake, weekly exercise frequency, parental weight status, community food outlet density. | TV 1-2h, >2h vs.<=1h: OR (95%CI) were 1.66 (1.33～2.31) , 2.01 (1.52～3.46) for overweight; 2 .26 (2.03～3.90) , 2.34 (2.18～2.72) for obesity . | TV <=1h/d | 13 | moderate |
| 48 | Dan Li | 2007 | Chinese | Cross-sectional | Shanghai | Grades 7-12 | 473 | 3-psychobehavioral problems | problematic behaviors | e-games | none | E-gaming time (> 30, 20-23, 10-20, 3-10, <3 h/week, none): problematic behavior score F (5, 441) =15.153, p <0.0001. | none | 8 | low |
| 49 | Wentao Yang | 2018 | Chinese | Cross-sectional | Beijing | Grades 4-6 | 456 | 1-adiposity | Overweight, obesity | TV | none | Students who had daily TV viewing > 2 vs. <=2h/d had greater body weight (41.9±9.9kg vs. 40.2±9.8kg, P=0.015) , higher BMI (18.7±3.9 kg/m^2 vs. 17.9±3.5 kg/m^2, P=0.026) , and higher overweight and obesity rate (22.7% vs. 16.5%, P=0.020) . | none | 6 | low |
| 50 | Peixian Ma | 1999 | Chinese | Cross-sectional | Guizhou | 14 years | 800 | 1-adiposity | Obesity | TV | none | TV viewing > 4 h/d vs. none: obesity rate 7.5% vs. 1.5%, χ2=4.08，P<0.01) . | none | 7 | low |
| 50 | Peixian Ma | 1999 | Chinese | Case-control | Guizhou Guiyang | 14 years | 800 | 2-myopia | Low vision | TV | none | TV >4h/d vs. <0.5h/d: myopia rates x^2=22.95，P<0.01. | none | 7 | low |
| 50 | Peixian Ma | 1999 | Chinese | Cross-sectional | Guizhou Guiyang | 14 years | 800 | 5-academic performances | exam scores | screen time | none | screen time >4h/d vs. <0.5h/d: exam scores (u=16.99，P<0.01). | none | 7 | low |
| 50 | Peixian Ma | 1999 | Chinese | Cross-sectional | Guizhou Guiyang | 14 years | 800 | 6-Sleep disorders | sleep disorders | TV | none | screen time >4h/d vs. <0.5h/d: sleep disorders (u=10.58，P<0.01). | none | 7 | low |
| 50 | Peixian Ma | 1999 | Chinese | Cross-sectional | Guiyang | 14 years | 800 | 10-Miscellaneous | Height | TV | none | TV viewing > 4 h/d vs. 0 h/d: 159.7 cm vs. 163.6 cm, t = 4.33, p < 0.01. | none | 7 | low |
| 50 | Peixian Ma | 1999 | Chinese | Cross-sectional | Guiyang | 14 years | 800 | 10-Miscellaneous | Sick leave rate | TV | none | Daily TV viewing > 4h/d vs. none, sickness leave rate: 75.25% vs. 21.25%, χ2 = 41.54,P<0.01. | none | 7 | low |
| 51 | Xiaojun Wang | 2012 | Chinese | Cross-sectional | Guangdong dongguan | Grade 10 | 546 | 2-myopia | Myopia | TV, e-games | eye exam, study pressue, reading hours | ST <1h vs. >=1h: myopia OR (95%CI) was 0.304 (0.102～0.901), P=0.031 | screen time <1h/d | 11 | moderate |
| 52 | Xiaojie Zhang | 2012 | Chinese | Case-control | Changchun | 7-18 years | 650 | 1-adiposity | Obesity | TV | none | Daily TV viewing time between children with obesity and normal weight: 2.10±1.16 vs. 1.76±0.34, t=4.97, p<0.001. | none | 9 | low |
| 53 | Zhong Chen | 2012 | Chinese | Cross-sectional | Hubei Wuhan | Grades 1-6 | 2061 | 6-Sleep disorders | sleep disorders | TV | none | weekday TV <1, 1-2, 2-3, >3h/d: prevalance of sleep disorders were 70.4%, 70.9%, 79.3%, 83.0%, x^2 = 10.32, p=0.016;  weekend TV <1, 1-2, 2-3, >3h/d: prevalance of sleep disorders were 65.7%, 68.1%, 73.2%, 78.1%, x^2 =14.93, p=0.002. | none | 8 | low |
| 54 | Mingming Wang | 2019 | Chinese | Cross-sectional | Shangdong Zibo | 6-11 years | 1319 | 4-cardiometabolic risks | left ventricular hypertrophy | TV, computer, e-games | gender, age, fruit and vegetable intake, sugary drink intake, physical activity, sleep hours, blood pressure | screen time >2h/d vs. <=2h/d: left ventricular hypertrophy OR(95%CI) was 2.12(1.08～4.32). After the additional adjustment of BMI, OR(95%CI) was 1.45(0.63～3.32). Weekday and weekend screen time were not significant. | screen time <=2h/d | 13 | moderate |
| 55 | Junxiang Liu | 2006 | Chinese | Cross-sectional | Chongqin | 3-16 years | 1867 | 1-adiposity | Obesity | TV | Parental weight status, birth weight, complementary food intake, breastfeeding, meat intake, vegetable intake, late sleep, picky eating, vitamin C supplement intake. | TV (h/d): obesity OR (95%CI) was 1.175 (1.033～1.335) . | none | 9 | low |
| 56 | Dongmei Wei | 2015 | Chinese | Cross-sectional | Beijing | 7-11 years | 1864 | 1-adiposity | BMI | TV, video, computer, games, iPad | age | BMI was positively associated with screen time (h/d) in boys (b = 0.27, P < 0.01) and in girls (b = 0.36, p < 0.01) . | none | 15 | high |
| 58 | Qing Tang | 2011 | Chinese | Cross-sectional | Guangxi Nanning | 7-18 years | 5658 | 1-adiposity | Obesity | TV, e-games | outdoor activity hours, fried food or sweets preference, feeding practices, birth weight, eating speed, parental weight status. | Screen time (<=1 h/d, 1-2 h/d, > 2 h/d, ordinal coding): obesity OR (95%CI) was 1.474 (1.339～1.624) . | none | 12 | moderate |
| 59 | Jian Wang | 2018 | Chinese | Cross-sectional | Shanghai | 9-14 years | 628 | 3-psychobehavioral problems | subjective well-being | TV; Internet; e-games | none | E-gaming was inversely associated with satisfaction with life (beta = -0.11, p < 0.01), personal well-being index (beta = -0.15, p < 0.01), and positively associated with negative affect (beta = 0.15, p < 0.01). | none | 10 | low |
| 60 | Chao Wang | 2016 | Chinese | Cohort | Beijing | Grades1-5 7-8 10-11 | 4003 | 2-myopia | Diopter progression | TV, computer | none | ST (h/d) was positively associated with diopter progress: Rs =0.05，P< 0.01. But null in hierarchical logistic regression. | none | 6 | low |
| 61 | Anyi Zhang | 2018 | Chinese | Cross-sectional | Eight cities | Grades 1-6 | 21980 | 1-adiposity | obesity | TV | number of friends, frequencies of disciplined by teacher and parents, physical activity | TV viewing time (<1,1-2,2-3,>3h/d, ordinal coding) , OR (95%CI) : 1.104 (1.035～1.178) . | none | 12 | moderate |
| 62 | Quan Zhou | 2014 | Chinese | Cross-sectional | Fofan | 7-17 years | 4583 | 1-adiposity | overweight and obesity | TV, computer | gender, age, picky eating, teeth brushing, sleep | Screen time >1h/d vs. <=1h/d: overweight and obesity OR (95%CI) was 1.398 (1.007～1.942) ，P<0.05. | screen time <=1h/d | 12 | moderate |
| 63 | Qiu Zhang | 2019 | Chinese | Cross-sectional | Fujian Fuzhou | Grades 1-12 | 1528 | 2-myopia | Low vision | eletronic devices | gender, outdoor activity, reading posture, reading light. | Screen time >=3h/d vs. <3h/d: myopia OR (95%CI) was 1.49 (1.26～1.77) , P<0.001. | screen time <3h/d | 14 | moderate |
| 64 | Xiaoyang Zhang | 2016 | Chinese | Cross-sectional | Fujian Fuzhou | 6-17 years | 1371 | 4-cardiometabolic risks | hypersention | TV; cellphone; computer, tablet | family history, waist circumference, BMI, staple intake, fruit and vegetable intake, diary intake, meat intake, walk-to-school time, outdoor activity | cellphone >=3.5h/w vs. <3.5h/w: hypertension OR(95%CI) was 1.888(1.200～3.385). TV >=14h/w vs. <14h/w: hypertension OR(95%CI) was 2.263(1.354～3.001). computer/tablet>=7h/w vs. <7h/w: hypertension OR(95%CI) was 2.121(1.462～3.173). | cellphone <3.5h/wk;  TV <14h/wk;  computer/tablet <7h/wk | 12 | moderate |
| 66 | Wenjuan Chen | 2011 | Chinese | Cross-sectional | Shanghai | Grades 1-6 | 2161 | 6-Sleep disorders | sleep disorders | computer | gender, age, parental education, family income, homework ours, bedtimelight, parental bedtime, athma, sleep with caregivers, recent life event, etc. | Computer >1h/d vs. <=1 h/d: sleep disorders OR(95%CI) were 2.122(1.462～3.079), P<0.001, | computer <=1 h/d | 13 | moderate |
| 68 | Hongjie Di | 2013 | Chinese | Cross-sectional | Gansu | Grades 1-6 | 2931 | 1-adiposity | overweight and obesity | TV, e-game | birth weight, picky eating, greesy food intake, sweets intake, indoor activity time, outdoor activity time, eating speed | TV viewing time and e-game time were both not associated with overweight and obesity rates. | none | 11 | moderate |
| 69 | Liangying Chen | 2019 | Chinese | Cross-sectional | Beijing, Shenzhen, Suzhou, Xiamen, Xiantao, Tongchen | Grades 6 and 8 | 840 | 1-adiposity | BMI | Online education | unknown | BMI was positively associated with online education hours in boys (b=0.072, p<0.05) but not in girls. | none | 10 | low |
| 70 | Weijia Liu | 2017 | Chinese | Cross-sectional | Guangdong Guangzhou | 7-12 years | 2859 | 7-poor physical fitness | 6-minute run, standing long jump | TV, computer or e-games | homework hours, gender, age , physical activity, vegetable intake, sugary drink intake , fast food intake, age of menarche, age of first spermatorrhea | weekday screen time >=2h/d vs.＜2h/d，6-minute run and standing long jump at the high athletic level OR (95%CI) were 0.68(0.51～0.89), 0.73(0.56～0.97).  Weekend 2h/d vs.＜2h/d, 6-minute run and standing long jump at the high athletic level OR (95%CI) were 0.95(0.96～1.48), 0.93(0.45～1.24) . | weekday and weekend screen time <2h/d | 11 | moderate |
| 71 | Caiqiong Zeng | 2018 | Chinese | Cross-sectional | Hubei | 6-10 years | 16955 | 2-myopia | Myopia | eletronic devices | grade level, parental myopia, outdoor hours, reading distance, study hours | ST (h/d, reverse coding): myopia OR=0.810, P<0.05. | none | 15 | high |
| 72 | Rong Lin | 2017 | Chinese | Cross-sectional | Guangzhou | 7-12 years | 9260 | 1-adiposity | overweight and obesity | TV, video, DVD; computer games | none | BMIz was positively associated with computer using time (Rs=0.048，P=0.002.), but was not associated with TV/DVD/video viewing time (Rs=0.002，p =0.915) . | none | 9 | low |
| 73 | Xiaohong Qi | 2017 | Chinese | Cross-sectional | Guangdong Guangzhou | Grades 3-9 | 3572 | 2-myopia | Low vision | TV, computer | grade level, homework hours, reading distance, reading while lying, nutrition status, dry eye. | ST <1h, 1-3h, >3h vs. none: myopia OR( 95%CI) were 1.059 (0.872～1.011) ，p= 0.147; 1.319 (1.121～1.412) ，p<0.001; 3.040 (1.138～5.273) ，p< 0.001. | screen time = 0 | 12 | moderate |
| 74 | Jingcheng Mai | 2010 | Chinese | Cross-sectional | Guangzhou | 7-18 years | 6708 | 1-adiposity | BMI | TV, e-games | sleep hours, sugary drink intake, PE preference, meat intake, weekend homeword load, diary intake, physical activity. | BMI was inversely associated with weekday TV/video viewing time (b=-0.131, 95% CI: -0.207～-0.055, p =0.001), but was not associated with computer using time and e-gaming time. | none | 9 | low |
| 74 | Jingcheng Mai | 2010 | Chinese | Cross-sectional | Guangdong Guangzhou | 7-18 years | 6708 | 2-myopia | Low vision | TV, e-games | sleep timing, sugary drink intake, meat intake, homework hours, physical activity. | myopia was positively associated with e-game time (b=0.013, 0.004～0.022, P=0.007) , but was not associated computer time and weekday TV/video time. | none | 9 | low |
| 74 | Jingcheng Mai | 2010 | Chinese | Cross-sectional | Guangdong Guangzhou | 7-18 years | 6708 | 7-poor physical fitness | fitness score | TV, computer, e-games | sleep time, sugary drink intake, meat intake, homework hours, diary intake, physical activity | physical fitness score were inversely associated with weekly computer using time b= -0.340(-0.540～-0.141), p=0.001, but was not significantly associated with TV viewing hours per day and e-game playing hours per day. | none | 9 | low |
| 75 | Weihao Huang | 2020 | Chinese | Cross-sectional | Guangzhou | Grade 7 , 10 | 12357 | 1-adiposity | overweight and obesity | Screen time | none | Overweight and obesity rates were higher between screen time groups >=2h/d vs. < 2h/d, χ2 = 25.08，P＜0.01. | none | 8 | low |
| 76 | Xiuqiong Feng | 2018 | Chinese | Cross-sectional | Guangdong Guangzhou | 8-18 years | 804 | 2-myopia | Myopia | TV; cellphone /tablets | gender, parental myopia, homework hours, reading while lying, reading while walking, sleep hours, dieting, commuting methods, outdoor time. | TV >2h/d vs. none: myopia OR (95%CI) was 2.52 (1.27～4.97) , P=0. 008. Cellphone and tablet: null. | TV =0 | 15 | high |
| 77 | Hui Wang | 2019 | Chinese | Cross-sectional | Guangzhou | 6-13 years | 4523 | 1-adiposity | central obesity | TV, computer, e-game | gender, age, only child, parental education , family monthly income, MVPA | screen time >2h/d vs. <=1 h/d: central obesity (OR (95% CI) was 1.43 (1.04～1.96) . | screen time <=1h/d | 15 | high |
| 77 | Hui Wang | 2019 | Chinese | Cross-sectional | Guangdong Guangzhou | 6-13 years | 4523 | 4-cardiometabolic risks | metabolic syndrome | TV, computer, e-games | gender, age, single child, parental education, family income, physical activity | screen time >2h/d vs. <=1 h/d: metabolic syndrome OR(95% CI) was 1.94 (1.11～3.40). | screen time <=1 h/d | 15 | high |
| 78 | Hongjie Li | 2017 | Chinese | Cross-sectional | Harbin | 9-18 years | 2658 | 1-adiposity | overweight and obesity | TV, e-games | sleep hours, daily homework hours, egg intake, PE class attendence, breakfast intake, parental physical activity. | Overweight and obesity rate was positively associated with screen time (b=0.11, P=0.03) | none | 13 | moderate |
| 79 | Yanhai Kang | 2013 | Chinese | Cross-sectional | Hainan Haikou | Grades 10-12 | 626 | 3-psychobehavioral problems | mental health | Internet | n.a. | Internet >10 h/wk vs. <=10h/wk, interpersonal sensitivity and psychotic OR (95% CI) were 2.346 (1.203～5.670), 2.636 (1.226～5.670). | internet <=10h/week | 11 | moderate |
| 80 | Yang Wang | 2015 | Chinese | Cross-sectional | Hebei Baoding | 7-18 years | 600 | 2-myopia | Myopia | TV, computer | none | Myopia group vs. normal group: daily ST time >2h were 28.2% vs. 18.1%, x^2 = 8.627, P=0.003. | none | 5 | low |
| 81 | Weifang Chen | 2014 | Chinese | Cross-sectional | Hebei Shijiazhuang | 7-18 years | 1796 | 2-myopia | Low vision | TV, computer | location of residence, reading posture, study hours, reading while lying, reading hours, physical activity , sleep hours, parental myopia. | ST 1h vs. <=1h/d: myopia OR (95% CI) was 1.276 (1.121～1.397) | screen time <=1h/d | 10 | low |
| 82 | Fengming Xu | 2017 | Chinese | Cross-sectional | Henan | 9-18 years | 34886 | 2-myopia | Low vision | TV; eletronic devices | age, gender, location of residence, sleep time, nutrition status, physical activity, etc. | TV 0.5-2h, >=2h vs. <0.5h/d, myopia OR (95% CI) was 0.887 (0.839～0.937) , 0.738 (0.681～0.799) . Non-TV ST 0.5-2h, >=2h vs. <0.5h: myopia OR (95% CI) was 1.020 (0.967～1.077) , 1.155 (1.072～1.244) . | TV <0.5h/d; non-TV screen time <0.5h/d | 14 | moderate |
| 83 | Xiaona Jia | 2019 | Chinese | Cross-sectional | Henan | 9-18 years | 34840 | 4-cardiometabolic risks | hypersention | TV; electronic devices | hypertension, BMI, sleep hours, dietary intake, physical activity, study load | electronic devices 1="<0.5 h/d", 2 =" 0.5～<2h/d"，3 = ">=2 h/d":hypertension OR (95% CI) was 1.09(1.04～1.14);  TV 1="<0.5 h/d", 2 =" 0.5～<2h/d"，3 = ">=2 h/d": hypertension p> 0.05. | none | 11 | moderate |
| 84 | Renqiang Chen | 2006 | Chinese | Cross-sectional | Shandong Heze | Grade 10-11 | 700 | 2-myopia | Myopia | computer, e-games, Internet | none | Myopia group vs. normal group: excessive screen time (14.02% vs. 7.17%, P<0.01) | none | 8 | low |
| 85 | Ailan Wang | 2015 | Chinese | Cross-sectional | Shandong Heze | Grades 1-12 | 29047 | 2-myopia | Myopia | computer; cellphone | outdoor activity, hand crafting activity, reading posture, study hours. | Computer (unknown coding): myopia OR (95% CI) was 10.751 (3.836～17.666) ;  Cellphone (unknown coding): myopia OR (95% CI) was 12.782 (6.370～19.193) . | none | 11 | moderate |
| 86 | Ran Hu | 2013 | Chinese | Cross-sectional | Heilongjiang | 16-18 years | 1398 | 2-myopia | Myopia | TV, computer | none | ST 2h/d vs. <=2h/d: myopia (71.31% vs. 55.6%) , x^2 = 22.833, p<0.05. | none | 7 | low |
| 87 | Wenli Hao | 2015 | Chinese | Cross-sectional | Inner mongolia | Grades 1-5 | 12384 | 2-myopia | Myopia | TV, e-games, Internet | gender, nationality, parental myopia, physical activity, eading posture, location of residence, grade level, parental occupation, etc. | Screen time (1= <1h，2= 1～2h，3= 2～3h，4=>3h), myopia OR (95%CI) was 1.08 (1.03～1.14) . | none | 12 | moderate |
| 88 | Shuqiong Hu | 2020 | Chinese | Cross-sectional | Hubei | 7-18 years | 10353 | 2-myopia | Myopia | eletronic devices | homework hours, outdoor time, reading posture,age | ST >2 vs. <=2h/d, myopia OR (95%CI) was 1.458 (1.331～1.598) . | screen time <=2h/d | 12 | moderate |
| 89 | Jiayu Cai | 2020 | Chinese | Cohort | Hubei | Grade 1 | 695 | 2-myopia | Myopia | eletronic devices | gender, premature birth, parental myopia, outdoor time, study hours, sweets and sugary drink intake. | ST 0.5h-1h, 1h-2h vs. <0.5h/d: myopia OR (95%CI) was 1.854 (1.039～3.308) , 2.960 (1.466～5.979) . ST >2 h/d vs. < 0.5h/d: myopia OR (95%CI) was 2.909 (0.495～17.095) . | screen time <0.5h/d | 14 | moderate |
| 90 | Lin Liu | 2016 | Chinese | Cross-sectional | Hunan Huaihua | Grades 3-12 | 2103 | 2-myopia | Low vision | TV, Internet | reading hours, reading posture, study desk condition, outdoor activity, watching distance, parental myopia, grade level, reading light, sleep hours, physical activity, after-school tutoring. | ST (reverse coding): myopia OR(95%CI) was 0.632 (0.461～0.867) . | none | 13 | moderate |
| 91 | Wudi Hao | 2019 | Chinese | Cross-sectional | Liaoning | Grades 1-6 | 876 | 2-myopia | Myopia | eletronic devices | nationality, parental myopia, outdoor and indoor physical activity, grade level, gender, sleep hours. | TV >=1h/d vs. <1h/d: myopia p>0.05, not included in the model. non-TV ST >=1h/d vs. <1h/d: myopia OR (95%CI) was 1.684 (1.012～2.801) . | TV <1h/d; non-TV screen time <1h/d | 14 | moderate |
| 92 | Xiaoyan Tan | 2012 | Chinese | Cross-sectional | Shandong | 6-12 years | 1584 | 1-adiposity | overweight and obesity | TV, computer | outdoor activity, sugary drink intake | TV and computer time (<1h, 1-2h, >=2h, ordinal coding): overweight and obesity OR (95%CI) was 1.788 (1.509-2.119) . | none | 11 | moderate |
| 93 | Yue Teng | 2020 | Chinese | Cross-sectional | n.a. | 6-12 years | 655 | 2-myopia | Myopia | Computer | gender, grade level, maternal myopia, reading hours, meat intake. | Computer >2h/time vs. <=2h/time，myopia OR (95%CI) was 2.16 (1.08～4.35) ，P=0.030. null after adjusting for confounding factors. | computer <=2h/time | 13 | moderate |
| 94 | Ganglei Pan | 2017 | Chinese | Cross-sectional | Zhejiang Ningbo | Grade 7-12 | 10727 | 2-myopia | Myopia | TV, e-games, eletronic devices | age, gender, parental myopia, physical activity, academic performance, health status, diary intake. | TV >=2h/d vs. <2h/d: myopia OR >0.05, not included in the model. Non-TV ST >=2h/d vs. <2h/d: myopia OR (95%CI) 1.170 (1.083 ~ 1.264). | TV <2h/d; non-TV screen time <2h/d | 13 | moderate |
| 95 | Sun G | 2016 | Chinese | Cross-sectional | Xuzhou | Grades 7-12 | 572 | 10-Miscellaneous | Injury | Internet | n.a. | Injury rate by weekly internet surfing time (none, <4h,4-10h,>10h): 23.9%, 35.6%, 44.3%, 37.3%, χ2 = 15.06, p = 0.002. Not significant in multilogistic regression model after adjusting for socio-demo-economic factors. | Internet 0h/wk | 10 | low |
| 96 | Jiali Duan | 2006 | Chinese | Cross-sectional | Beijing | Grades 1-12 | 7362 | 2-myopia | Low vision | TV | location of residence, grade level, gender, reading distance, reading posture, study hours, sleep hours. | Daily TV viewing time: myopia OR (95%CI) was 0.908 (0.852～0.967) . | none | 12 | moderate |
| 97 | Zhili Guo | 2016 | Chinese | Cross-sectional | Zhejiang Jiaxing | Grade 1 | 1068 | 2-myopia | Myopia | computer, iPad, cellphone, etc. | family myopia, premature birth, reading distance reading posture, homework hours, recreational hours. | non-tv ST ＞3 vs. <=3h/d: myopia beta=0.19, OR=17.81，P=0.02. | screen time <=3h/d | 12 | moderate |
| 98 | Yi Fan | 2018 | Chinese | Cross-sectional | Jiangxi | Grade 7-12 | 4826 | 2-myopia | Myopia | TV; computer; cellphone, tablets | unknown | Cell/tablet (5-point likrt scale reverse coding): OR (95%CI) was 0.49 ( 0.32～0.74) , 0.52 (0.35～0.78) , 0.62 (0.43～0.91), 0.60 (0.41～0.86) .  TV and computer time were not significantly associated with myopia. | none | 7 | low |
| 100 | Tingting Ren | 2018 | Chinese | Cross-sectional | Kashi, Xinjiang | 7-18 years | 2644 | 1-adiposity | overweight and obesity | Screen time | gender, grades, school, LPA, MVPA | screen time ＞3h/d, 2～3h/d vs. ＜2h/d: overweight and obesity OR (95%CI) were 2.33 (1.60～ 3.19) and 1.81 (1.12～ 2.96), p < 0.05. | screen time <2h/d | 14 | moderate |
| 101 | Bing Zhang | 2014 | Chinese | Cross-sectional | Ningbo | 11-17 years | 1667 | 1-adiposity | Obesity | internet | none | Internet >3 h/d vs. <= 3 h/d: obesity rates were not different (c2 = 2.12，p = 0.126) . | none | 9 | low |
| 101 | Bing Zhang | 2014 | Chinese | Cross-sectional | Zhejiang Ningbo | 11-17 years | 1667 | 2-myopia | Myopia | Internet | none | Internet >3h/d vs. <=3h/d: myopia (x^2 = 10.75，p = 0.001) . | none | 10 | low |
| 102 | Xueqin Xiong | 2019 | Chinese | Cross-sectional | Szechwan | 6-12 years | 868 | 3-psychobehavioral problems | social competence and problematic behaviors | TV, computer, cellphone | age, gender, location of residence, parental invovlement, age of screen use | screen time was inversely associated with social competence score in boys (beta = -1.121, p = 0.004)and girls (beta = -1.074, p = 0.002).and positively associated with social withdrawal (beta = 0.119, p = 0.002), aggressivenes (beta = 0.04, p = 0.033), compulsivity (beta = 0.008, p = 0.002) in boys, and imaturity (beta = 0.019, p = 0.004) in girls. | none | 11 | moderate |
| 103 | Yue Wu | 2019 | Chinese | Cross-sectional | Shangdong Zibo | 12-18 years | 532 | 4-cardiometabolic risks | pre-diabetes | TV, cellphone, computer | gender, age, BMI, systolic pressure, diastolic pressure , domestic residency, smoking and drinking, energy-dense food intake,sugary drink intake, physical activity, gestational diabetes,premature birth, birth weight, family history of diabetes | screen time 120-239min/d, 240～317/d, >=318 min/d vs. <=119min/d, pre-diabetes were OR(95%CI)1.059(0.515～2.180), 2.502(1.279～4.897), 2.337(1.189～4.594), p = 0.876, 0.007, 0.014 | screen time <=2/d | 16 | high |
| 104 | Yuehui Jia | 2019 | Chinese | Cross-sectional | Tsitsihar | 7-13 years | 2380 | 1-adiposity | overweight and obesity | Screen time | gender, age, sleep hours, breakfast intake, physical activity, homework hours, parental attitudes toward physical activity | screen time (none, <0.5, 0.5 ~<1.0, 1.0 ~<2.0, 2.0 ~<3.0, >=3.0, ordinal coding): overweight and obesity rate OR (95%CI) was 4.90 (2.62, 9.18) . | none | 14 | moderate |
| 105 | Zhaoxuan Zheng | 2019 | Chinese | Cross-sectional | Shandong Qingdao | Grades 10-12 | 2673 | 2-myopia | Myopia | screen time | grade level, outdoor hours, familial myopia, healthy literacy of myopia. | ST 0.5-1h/d, 1-1.5,1.5-2,>2 vs.<=0.5h/d: myopia OR (95%CI) were 1.126 (0.842～1.603), 1.455 (1.007～2.103) , 1.508 (1.109～2.051) , 1.720 (1.212～2.442). | screen time <=0.5h/d | 16 | high |
| 106 | Wenping Li | 2020 | Chinese | Cross-sectional | 15 aviation schools | 14-19 years | 1286 | 3-psychobehavioral problems | mental health | cellphone | none | Cellphone use (h/d) had a inverse relationship with psychological flexibility (Z=－0.142, P<0.001). | none | 6 | low |
| 107 | Su Liu | 2019 | Chinese | Cohort | Hubei Wuhan | Baseline 10-15 | 869 | 5-academic performances | exam scores | screen time | gender, age, grade, study load, physical activity | exam scores was positively associated with screen time (b=-0.29, p<0.01). | none | 9 | low |
| 108 | Liu R | 2011 | Chinese | Cross-sectional | An Hui. Zhidong County | 9-18 years | 5407 | 10-Miscellaneous | Delayed puberty | TV | none | Daily TV viewing time between girls who had delayed and normal puberty: 78.48 ± 99.43 vs. 83.26 ± 106.26 min/d, p > 0.05.  Daily TV viewing time between boys who had delayed and normal puberty: 80.64 ± 97.18 vs. 94.22 ± 112.22 min/d, p < 0.05. | none | 9 | low |
| 109 | Zhiying Miao | 2015 | Chinese | Cross-sectional | Jinan | 11-23 years | 2137 | 9-sub health | Physical and mental sub-health | Internet | regular breakfast, physical activity, habit of siesta | Internet surfing >=4h/d vs. <4h/d OR (95% CI): 20.95 (5.50, 79.82). | Internet <4h/d | 12 | moderate |
| 110 | Zhuoya Liu | 2012 | Chinese | Cross-sectional | Hubei | 9-18 years | 3182 | 1-adiposity | overweight and obesity | TV, internet | gender, age, family structure, sleep | Internet >=2h/d vs. <2h/d: overweight and obesity OR (95%CI) was 1.393 (1.031～1.883) , p=0.031. TV >=2h/d vs. <2h/d: not significant. | Internet <2h/d; TV <2h/d | 14 | moderate |
| 111 | Fang Cheng | 2017 | Chinese | Cross-sectional | Zhejiang Ningbo | 12-21 years | 2218 | 3-psychobehavioral problems | Attention deficiency | TV | gender, age, single child, academic performance, insomia, daytime drowsiness | TV ("<1 h/d" = 1, "1～2 h/d" = 2, ">=3 h/d" = 3) : attention deficiency OR (95%CI) was 1.458 (1.015～2.095). | TV < 1h/d | 12 | moderate |
| 112 | Ruiru Liu | 2011 | Chinese | Cross-sectional | Shanxi | Grades 7-12 | 814 | 3-psychobehavioral problems | problematic behaviors | e-games | none | E-games 0 times, 1-2 times, 3-4 times and >4 times/week: study resistance <0.05. | none | 9 | low |
| 113 | FengningSong | 2005 | Chinese | Cross-sectional | Guangxi Nanlin Guilin | Grades 10-11 | 426 | 3-psychobehavioral problems | pro-social behaviors | Internet | none | Internet <=2, 2-6, >6h/week: sharing (F = 5.04, p <0.01) and cooperation (F = 3.44, p <0.05). | none | 8 | low |
| 114 | Jing Ding | 2015 | Chinese | Cross-sectional | Fujian Ningde | Grades 2-6 | 345 | 3-psychobehavioral problems | cognitive capacity | Mobile devices | none | Mobile devices using time and cognitive score: Rs=-0.133, p<0.05). | none | 6 | low |
| 115 | Weiyan Gao | 2019 | Chinese | Cross-sectional | Ningxia | Grades 1-12 | 1936 | 2-myopia | Low vision | TV | reading posture, reading light | TV (1 = "<1h/d"; 2 = "1～2h/d"; 3 = "2～3h/d"; 4 = ">=3h/d"; 5 = "unknown"): myopia OR=1.166, p < 0.05. | none | 10 | low |
| 116 | Zhenpeng Xue | 2020 | Chinese | Cross-sectional | Shenzhen | 9-20 years | 1751 | 9-sub health | Physical and mental sub-health | Internet | interpersonal relationship, age, gender, single child, parental marrige, academic pressure | Daily time spent on internet surfing: β = -0.126，P<0.05 for scores of physical and mental health assessment. | none | 10 | low |
| 117 | Bizhen Luo | 2015 | Chinese | Cross-sectional | Guangdong Ruyuan | Grades 1-9 | 1146 | 2-myopia | Low vision | screen time | none | ST >=1h vs. <1h: myopia (x^2 = 15.86，P<0.01) . | none | 8 | low |
| 118 | XiaotongWen | 2019 | Chinese | Cross-sectional | Jiangxi Nanchang | 14-18 years | 844 | 1-adiposity | overweight and obesity | TV | gender, age, weight management behaviors, sugary drink intake, animal-based food intake, vegetable intake, fruit intake, breakfast inake, physical activity, sleep hours, etc. | TV viewing >= 1 h/d vs. < 1 h/d : overweight and obesity OR (95%CI): 1.19 (1.09～3.34) , p=0.023 . | TV <1h/d | 10 | low |
| 119 | Shaoqian Lin | 2012 | Chinese | Cross-sectional | Rural Shandong | 7-12 years | 990 | 1-adiposity | overweight and obesity | TV | sleep hours, fried food intake, parental weight status, birth weight | TV >2h/d vs. <=2h/d: overweight and obesity OR (95%CI) was 3.062 (1.708～4.932). | TV <=2h/d | 13 | moderate |
| 120 | Na Zhao | 2019 | Chinese | Cross-sectional | Shanxi | 15-18 years | 900 | 4-cardiometabolic risks | hypersention | cellphone | posture, BMI, etc. | cellphone >6h vs. <=6h (unknown timeframe): hypertension(P=0.005,OR(95%CI) was 1.900(1.218～2.965). | cellphone <=6h | 15 | high |
| 121 | Jun Zhang | 2013 | Chinese | Cross-sectional | Shanghai | 7-9 years | 2234 | 1-adiposity | Obesity | TV | gender, age, appetite, eating speed, sleep hours, PE liking, meat intake, fish and seafood intake. | TV 1-2h, >=2 h vs. <=1h/d : obesity OR (95%CI) were 1.502 ( 1.134～1.988) and 1.431 ( 0.964～2.124) , p<0.05. | TV <=1h/d | 11 | moderate |
| 123 | Bing Wang | 2015 | Chinese | Cross-sectional | Shanghai | 8-11 years | 651 | 1-adiposity | Obesity | TV, computer | none | TV and computer using time was not associated with overweight and obesity, not included in the multiple logistic regression. | none | 13 | moderate |
| 124 | Chunyan Luo | 2011 | Chinese | Cross-sectional | Shanghai | 11-16 years | 2549 | 1-adiposity | Overweight, obesity | Computer, gaming device, cellphone, e-reader | none | screen time was different among obese, overweight, normal weight groups: 3.25, 3.03, 2.74 h/d, χ2 = 13.380，P<0．05. obese vs.overweight: no significant difference.  Obese vs. normal weight: χ2 =8.486, p = 0.004;  overweight vs. normal weight: χ2 =3.271, p = 0.0710. | none | 8 | low |
| 126 | Danfeng Zhang | 2016 | Chinese | Cross-sectional | Shanghai | 9-17 years | 3295 | 2-myopia | Myopia | computer, cellphone, e-games | none | ST was not associated with myopia, not added in the model. | none | 13 | moderate |
| 127 | Qin Huang | 2017 | Chinese | Cross-sectional | Jiangsu Nanjing | Grades 4 and 6 | 1199 | 2-myopia | Myopia | TV; screen time | age, sleep time | TV and myopia: b=-0.093, 95%CI (-0.050～-0.010) , p = 0.003.  Non-TV ST and myopia: b=0.067, 95%CI (0.002～0.040) , p = 0.033. | none | 10 | low |
| 129 | Maolin Yang | 2019 | Chinese | Cross-sectional | Shanghai | 7-13 years | 3894 | 1-adiposity | Obesity | Screen time | none | obesty rate was not associated with screen time. | none | 11 | moderate |
| 130 | Dongling Yang | 2015 | Chinese | Cross-sectional | Shanghai | 7-12 years | 1511 | 1-adiposity | BMI | electronic devices | gender, age, meal size, physical activity, sleep hours | overwegiht and obestiy was not assciated with screen time. BMI was associated with weekday computer time (b= 1.03，P<0.01) . | none | 12 | moderate |
| 131 | Shenghui Li | 2009 | Chinese | Cross-sectional | Shanghai | Grades 1-5 | 4108 | 6-Sleep disorders | sleep disorders | TV | gender, age, BMI , parental age, parental education, family income, family type. | weekday TV >=2h/d vs. <2h/d: unhealthy bedtime habits, irregular sleep hours, sleep disorders OR(95%CI) were 1.65(1.14～2.38), 1.58(1.05～2.37), 1.52(1.04～2.23). weekend >=2h/d vs. <2h/d: unhealthy bedtime habits, irregular sleep hours, sleep disorders OR(95%CI) were 1.35(1.17～1.57), 1.27( 1.06～1.52), 1.38( 1.19～1.59), 1.34( 1.16～1.55) | weekday and weekend TV <2h/d | 13 | moderate |
| 132 | Yang Yang | 2019 | Chinese | Cross-sectional | Shanghai | 9-18 years | 11913 | 1-adiposity | Overweight, obesity | TV, e-games, computer | sleep hours, breakfast intake, diary intake, egg intake, PE class frequency and preference, sports attitudes | TV 1-2h/d, >=2h/d vs. <1h/d: overweight and obesity OR (95%CI) were 1.22 (1.05～1.43) , 1.17 (0.91～01.51), remain significant in urban and rural sub samples, not significant by school levels. computer and e-gaming time 1-2h/d, >=2/d vs. < 1h/d: overweight and obesity OR (95%CI) was 1.04 (0.93～1.18) , 1.03 (0.88～1.21) ,not significant in urban and rural sub-samples and inconsistent by school levels. | TV <1 h/d; computer and e-games <1 h/d | 11 | moderate |
| 133 | Wang Fang | 2012 | Chinese | Cross-sectional | Guangdong shenzhen | 4-17 years | 3771 | 2-myopia | Myopia | TV, computer | age, reading distance, outdoor time, reading distance | TV and myopia: p> 0.05, not included in the model.  Computer >=1h/d vs. <1h: myopia OR(95% CI) was -0.148 (-0.052 ～-0. 041), p < 0.001. | computer <1h/d | 12 | moderate |
| 134 | Xian Gao | 2018 | Chinese | Cross-sectional | Beijing, Shanghai, Nanjing, Xi'an | Grades 1-12 | 1982 | 1-adiposity | Obesity | Leisure screen time | none | Students with obesity had greater leiture screen time than counterparts (χ2 = 13.380, P<0.05) | none | 10 | low |
| 135 | Meihao Gao | 2012 | Chinese | Cross-sectional | Shenzhen | 7-14 years | 8193 | 3-psychobehavioral problems | ADHD | TV | birth asphyxia, picky eating, left handness, maternal occupation, classmate relationship | TV (1= "<=1h/d", 2="1-2h/d", 3="2-4h/d", 4=">4h/d"), ADHD OR (95%CI) was 0.727 (0.652～0.810). | none | 11 | moderate |
| 136 | Lin Gong | 2006 | Chinese | Case-control | Shenzhen | 6-9 years | 479 | 10-Miscellaneous | Precocious puberty | TV | none | Daily TV viewing time <= 2 vs. > 2 h/d: 36.8 % vs. 16.5%, χ2 = 10.11, p = 0.001 | none | 11 | low |
| 137 | Yan Wang | 2014 | Chinese | Cross-sectional | Shenzhen | 7-13 years | 311 | 1-adiposity | overweight and obesity | TV or Computer | none | TV or computer time: >1h, 0.5-1h, <0.5h vs. none: overweight and obesity rate ( χ2 = 8.32，P=0.03). | none | 9 | low |
| 138 | Dingyan Shen | 2020 | Chinese | Cross-sectional | Guangdong shenzhen | Grades 1-12 | 3037 | 2-myopia | Myopia | TV or e-games | school recess, reading distance, PE classes, parental myopia | TV: myopia OR > 0.05, not included in the model.  Computer 2-3h/d vs. none: myopia OR (95%CI): 2.23 (1.19～4.20) ，P=0.01 in primary schoolers, null in junior and senior middle schoolers. | computer = 0. TV = 0. | 13 | moderate |
| 139 | Li Zhou | 2018 | Chinese | Cross-sectional | Shenzhen | Grades 7-12 | 3952 | 8-musculoskeletal injuries | spine health | cellphone; TV; computer | gender, school type, sleeping on desk during lunch break, physical activity, stay up late, study load, prolonged homework time | cellphone >40 min/time (sometimes, often vs. never) shoulder and neck discomforts OR(95% CI) were 1.317(0.985～1.761), 2.088(1.542～2.828)，waist and back discomforts OR(95% CI) were 1.113(0.815～1.519), 1.662(1.198～2.305);  TV>40 min/time (sometimes, often vs. never) shoulder and neck discomforts OR(95% CI) were 0.834(0.656～1.060), 1.220(0.934～1.594)，waist and back discomforts OR(95% CI) were 0.912(0.697～1.192), 1.588(1.182～2.133);  computer>40 min/time (sometimes, often vs. never) shoulder and neck discomforts OR(95% CI) were 1.069(0.822～1.391), 1.376(1.037～1.827)，waist and back discomforts OR(95% CI) were 0.912(0.697～1.192), 1.588(1.182～2.133). | none | 12 | moderate |
| 140 | Lan Wang | 2020 | Chinese | Cross-sectional | Liaoning Shenyang | Grades 7-12 | 775 | 3-psychobehavioral problems | self-injury | TV, cellphone, computer, tabletcomputer | health literacy, gender, grade level | weekend ST >=2h/d vs. < 2h/d: non-suicidal self-injury OR (95%CI) was 1.74 (1.09～2.78).  Weekday ST >=2h/d vs. < 2h/d: p > 0.05. | weekday and weekend screen time < 2h/d | 11 | moderate |
| 141 | Jin Wang | 2019 | Chinese | Cross-sectional | Liaoning Shenyang | 12-20 years | 1062 | 3-psychobehavioral problems | depression | TV, cellphone, computer, tabletcomputer | health literacy, interpersonal relationship, stress management, grade level, family income | ST>2h/d vs. <=2h/d, depressive symptoms OR (95%CI) was 1.431 (1.027～1.994). | screen time <=2h/d | 14 | moderate |
| 142 | Manran Yu | 2015 | Chinese | Cross-sectional | Liaoning | n.a. | 1712 | 2-myopia | Myopia | PAD or cellphone, computer | gender, parental myopia, study hours, outdoor time, reading distance, reading hours, sleep hours, reading posture, preferences for sweets. | Non-TV ST 1-2h/d,>=2h/d vs. <=1h/d: myopia OR (95%CI) was 1.176 (1.033～1.942) , 3.059 (1.008～8.450) ). | non-TV screen time <=1h/d | 12 | moderate |
| 143 | Lin Zhang | 2019 | Chinese | Cross-sectional | Liaoning Shenyang | 7-14 years | 1760 | 1-adiposity | Obesity | TV, internet | sweet food preference, fried food preference, physical activity, parental weight status | TV/internet time >=2h/d vs. < 2h/d: obesity rate OR (95%CI) was 2.114 (1．398～3．913) | screen time <2h/d | 12 | moderate |
| 144 | Zhaoxia Nian | 2018 | Chinese | Cross-sectional | Liaoning | 7-16 years | 1780 | 2-myopia | Myopia | computer, cellphone | age, reading distance, reading light, parental myopia, sleep with lights on. | Non-TV ST >= 3 h/d vs. < 3h/d: myopia OR (95%CI) was 1.32 ( 0.87～2.16) . | non-TV screen time <3h/d | 14 | moderate |
| 145 | Xifen Zhang | 2014 | Chinese | Cross-sectional | Heibei Shijiazhuang | Grades 7-12 | 2000 | 8-musculoskeletal injuries | spine subhealth | cellphone; computer | neck injury, sphagitis，severe myopia, life pressure, stay up late，reading while lying, pillow height, physical activity, sitting posture, reading distance, reading time | computer >4h/d vs. <=4h/d): spine sub-health OR(95%CI) was 4.767(3.000～7.577);  cellphone >4h/d vs. <=4h/d: spine sub-health OR(95%CI) was 3.676(2.303～5.868). | computer <=4h/d; cellphone <=4h/d | 10 | low |
| 146 | Haichao Zhang | 2017 | Chinese | Cross-sectional | Hebei Shijiazhuang | Grades 5-6 | 770 | 1-adiposity | obesity | TV or video;  e-games;  internet. | dietary intake, physical activity | Obesity rate was not associated with screen time ( 0, 1-2, and >=3 h/d). | none | 13 | moderate |
| 147 | Xiaoyue Wang | 2018 | Chinese | Cross-sectional | Szechwan | 6-14 years | 13116 | 2-myopia | Myopia | eletronic devices | reading distance, reading while walking and lying, reading light, inadequate sleep. | ST 1h vs.<=1h: myopa OR (95%CI) was 1.873 (1.258～4.785) . | screen time <=1h/d | 8 | low |
| 148 | Jie Wang | 2007 | Chinese | Cross-sectional | Jilin | Grades 10-12 | 5134 | 8-musculoskeletal injuries | cervical illness | computer, TV | study load, reading posture, physical activity, etc. | screen time (unknown coding): cervical illness OR=1.074, p=0.026. | unclear | 8 | low |
| 149 | Liuwei Zheng | 2018 | Chinese | Cross-sectional | Szechwan | Grade 11 | 2062 | 5-academic performances | poor exam scores | internet | none | students with low vs. high exam scores: percentages of weekend screen time >=4h ( 43.7% vs. 25.0%, x^2 =44.40，p< 0.001).  students with low vs. high exam scores: percetages of weekday screen time >=2h (22.4% vs. 31.9%, x^2=29.79，p< 0.001). | none | 7 | low |
| 150 | Wei Shi | 2017 | Chinese | Cross-sectional | Hebei Tangshan | 7-18 years | 7120 | 2-myopia | Myopia | computer | age, parental myopia, reading light. | Computer >3h/d vs. <=3h/d) : myopia OR (95%CI) was 1.327 (1.463～2.135). | computer <=3h/d | 13 | moderate |
| 151 | Yuting Wang | 2019 | Chinese | Cross-sectional | Tianjin | Grades 7-12 | 4127 girls | 10-Miscellaneous | Age of menarche | Electronic devices | none | No difference in age of menarche were found by daily TV viewing time, p = 0.14. | none | 8 | low |
| 152 | Jinhong Shi | 2016 | Chinese | Cross-sectional | Tianjin | 7-22 years | 9607 | 1-adiposity | overweight and obesity | TV, e-games | sleep hours, breakfast intake, diary intake, egg intake, PE class frequency and preference, sports attitudes | TV >=1h/d vs. < 1h/d: overweight and obesity OR (95%CI) was 1.366 ( 1.196～1.560) .  E-gaming 1-2h/d, >=2h/d vs. <1h/d: overweight and obesity OR (95%CI) were 0.867 (0.737～1.020) , 0.942 (0.787～1.129) . | TV <1h/d; E-gaming <1h/d | 13 | moderate |
| 153 | Xiaopeng Dong | 2018 | Chinese | Cross-sectional | Tianjin | Grades 1-6 | 1404 | 2-myopia | Low vision | computer; cellphone; tablet | grade level, gender, using eletronic devices in dim light. | Cellphone >=1.0h/d vs. <1h/d: myopia OR (95%CI) was 1.458 (1.036～2.051) .  Computer: myopia p >0.05. Tablet: myopia p >0.05. | cellphone <1h/d | 14 | moderate |
| 154 | Hongjing Zhou | 2010 | Chinese | Cross-sectional | Guangdong | 15-18 years | 838 | 3-psychobehavioral problems | mental health | Internet | none | Internet >2 h/week vs. <=2h/week: SCL-90 score 1.98 vs. 1.60, t = 2.889, p < 0.001. | none | 8 | low |
| 155 | Yafei Wu | 2011 | Chinese | Cross-sectional | Shanxi | Grades 1-6 | 2683 | 2-myopia | Myopia | computer | age, watching distance,age, parental education, reading light, reading posture, study desk condition. | Computer (1="<=1 hour/time"，2=">=1hour/time")，myopia OR (95%CI) was 1.899 (1.6143～2.2354). | computer < 1h/time | 13 | moderate |
| 156 | Yan Kong | 2012 | Chinese | Cross-sectional | Zhejiang Wenzhou | Grades 1-6 | 3207 | 2-myopia | Myopia | computer | parental myopia, watching distance, long reading hours, total study hours, etc. | Computer (＜"0. 5h/d" = 1，"0.5～ 1h/d"= 2，"1～2h/d" = 3, ">=2h/d" = 4) , myopia OR (95%CI) was 1.338 (1.105～1.620). | none | 14 | moderate |
| 157 | Mei Peng | 2020 | Chinese | Cross-sectional | Jiangsu | Grades 1-9 | 1072 | 3-psychobehavioral problems | Social adaptability | Internet | left-behind child, frequency of communicating with parents, life attitude, parenting style, etc. | Internet >=0.5h/d vs. none: social adaptability (b=0.11, p<0.05). Internet >=2h/d vs. none: social adaptability (b=-0.10, p<0.05). | internet = 0 | 11 | moderate |
| 158 | Meiling Qian | 2018 | Chinese | Cross-sectional | Linxia Zhou | 6-18 years | 8683 | 2-myopia | Low vision | eletronic devices | gender, age, study hours, watching distance, reading distance, outdoor time, study pressure, academic perfornace, light polution, unhealthy eye-use habits, reading light, preference for sweets, spicy foods, vegetables, and meat. | Weekend ST (uknown coding) myopia OR (95%CI) was 1.738 (1.197～2.522) .  Weekday TV (unknown coding): myopia >0.05, not included in the model. | none | 13 | moderate |
| 159 | Yahong Li | 2018 | Chinese | Cross-sectional | Gansu | 7-18 years | 4200 | 1-adiposity | overweight and obesity | TV, eletronic devices | location, gender, age, sleep hours, frequency of breakfast intake, milk intake, egg intake, academic burden | TV viewing time >0.5 h/d vs.=<0.5h/d: overweight and obesity OR (95%CI) was 1.14 (0.92～1.41) . E-game time >=3h/d vs.<3h/d: overweight and obesity OR (95%CI) was 0.87 (0.43～1.77) . | TV <=0.5h/d; E-games <3h/d | 14 | moderate |
| 160 | Chunli Li | 2017 | Chinese | Cross-sectional | Gansu Lanzhou | 6-20 years | 2354 | 2-myopia | Myopia | computer | height, weight, study hours, sleep hours, outdoor time. | Computer (0-1，1-2，2-3，3-4，>4h/d, ordinal coding)，myopia OR (95%CI) was 1.104 (1.032～1.182) . | none | 13 | moderate |
| 161 | Fangbiao Tao | 2004 | Chinese | Cross-sectional | Anhui Hefei | 13-20 years | 3127 | 3-psychobehavioral problems | depression and anxiety | Internet | gender , age , grade level , location of residence, school type, maternal education | Excessive internet surfing (unknown coding): depression and anxiety OR (95%CI) were 1.619 (1.126～2.327) and 1.616 (1.061～2.462). | unknown | 12 | moderate |
| 162 | Baocheng Zhu | 2014 | Chinese | Cross-sectional | Shenyang, Xinxiang, Guangzhou, Chongqin | Grades 7-12 | 13817 | 9-sub health | Physical and mental sub-health | TV, Cellphone, MP4, DVD/VCD | location, gender, school grade, parental education, family income, smoking and drinking behaviors | Weekday screen time >2h/d vs. <= 2h/d OR(95%CI): 1.39(1.25～1.55) for physical sub-health, 1.62(1.45～1.81) for mental sub-health, and 1.59(1.43～1.78) for physical and mental sub-health. Weekend day screen time >2h/d vs. <= 2h/d OR(95%CI): 1.49 (1.37～1.61) for physical sub-health, 1.81 (1.66～1.98) for mental sub-health, and 1.71 (1.57～1.87) for physical and mental sub-health. Weekday and weekend screen time both >2h/d, one >2h.d vs. both <= 2h/d OR(95%CI): 1.87 (1.65～2.12) and 1.38 （1.26 ~ 1.51）for physical sub-health, 2.47 (2.17～2.82) and 1.68 (1.53 ~ 1.85) for mental sub-health, 2.35 (2.06～2.68) and 1.57(1.43, 1.72) for physical and mental sub-health. | ST <=2h/d; ST weekday and weekend <=2h/d | 11 | moderate |
| 163 | Zhirong Zhang | 2018 | Chinese | Cross-sectional | Tibet, Shanxi, Guangzhou | Grades 7-12 | 3026 | 3-psychobehavioral problems | mental health | Internet | sleep hours, academic burden, physical activity, drinking, picky eating, number of friends | Internet >=4h/d, sub-mental health OR (95%CI) was 2.298 (1.299～4.067). | unknown | 9 | low |
| 164 | Damao Zhang | 2014 | Chinese | Cross-sectional | Zhejiang Ningbo | Grades 1-9 | 1280 | 2-myopia | Myopia | TV, computer | reading distance, parental myopia, homework hours, physical activity. | ST (0="<1h/d",1="1-2h/d",2=">3h/d") , myopia OR (95%CI) was 2.845 (1.383～5.216). | none | 10 | low |
| 166 | Duru Cai | 2005 | Chinese | Case-control | Guangdong shenzhen | Grades 1-9 | 1562 | 2-myopia | Myopia | TV, computer | none | ST >=2h/d vs. <2h/d: myopia rates x^2= 59.66， p<0.001. | none | 7 | low |
| 167 | Xiaoqin Hu | 2015 | Chinese | Cross-sectional | Shaoxing | Grades 1-9 | 3560 | 2-myopia | Myopia | computer | age, reading light, reading distance, reading posture, reading hours, etc. | Computer＞2h vs. <=2h/d, myopia OR (95%CI) was 1.216 (1.352～1.924). | computer <=2h/d | 10 | low |
| 168 | Weikang Yang | 2016 | Chinese | Cross-sectional | Guangdong shenzhen | 6-18 years | 3229 | 2-myopia | Myopia | TV, computer; cellphone | grade level, parental education, academic performance, sleep hours. | TV and computer (>=1h/d vs. < 1h/d): myopia OR (95%CI) was 1.355 (1.172～1.566) . Mobile devices (>=1h/d vs. < 1h/d): myopia OR (95%CI) was 1.355 (1.172～1.566) . Both came insignificant after adjusting for covariates. | TV and computer < 1h/d; Mobile devices < 1h/d; | 12 | moderate |
| 169 | Yuefang Zhang | 2015 | Chinese | Case-control | Xi'an | 11-12 years | 1535 | 1-adiposity | Obesity | TV, e-games | none | TV and e-games time >=2h/d vs.<2h/d: obesity rates between groups X^2=7.09 P=0.01 | none | 10 | low |
| 170 | Hanqiong Hu | 2014 | Chinese | Cross-sectional | Hangzhou | Grades 7-9 | 1139 | 1-adiposity | Obesity | leisure screen time; educational screen time | dietary restriction, physical activity, sleep, academic burden | Weekend leisure screen time > 3h vs.<= 3h: obesity OR (95%CI) was 2.227 (1．439～3．445) in boys, 1.923 (1.034～3．577) in girls. Weekend educational screen time > 50min vs. < = 50 min: obesity OR > 0.05 in boys, 2.073 (1.094～3.928) in girls. | weekend leisure screen time<= 3h/d; weekend educational screen time< = 50 min | 11 | moderate |
| 171 | Dongling Yang | 2016 | Chinese | Cross-sectional | Shanghai | Grades 1-5, 7-8, 10-11 | 9583 | 2-myopia | Myopia | TV; computer/e-games | gender, age, parental myopia, homework hours, physical activity | myopia was not associated with TV (h/d) and computer and e-games (h/d). | none | 14 | moderate |
| 172 | Meiqin Hu | 2009 | Chinese | Cross-sectional | Xi'an | Grades 7-12 | 737 | 1-adiposity | overweight and obesity | TV; Computer; e-games | gender, grades, location, parental education | TV >= 2h/d, 1-2h/d vs. <1 h/d: overweight and obesity OR (95%CI) were 1.89 (1.00～3.59) and 1.27 (0.63～2.57) . Computer >= 0.5h/d vs. <0.5 h/d: overweight and obesity OR95%CI) was 1.49 (0.93～2.39) . E-gaming >=0.5h/d vs. <0.5 h/d: overweight and obesity OR (95%CI) was 1.47 (0.90～2.39) . | TV < 1 h/d; computer < 0.5h/d; e-games <0.5h/d | 13 | moderate |
| 173 | Xin Huang | 2016 | Chinese | Cross-sectional | Yunnan | 13-18 years | 3594 | 7-poor physical fitness | fail in fitness exam | TV, computer, e-games | gender, location of residency, family income, nutrition status | screen time ("<=2 h" = 1，">2 h" = 2), fail rates in physical fitness exam OR(95%CI) were 1.805( 1.222～ 2.665) in 13-15-year-olds, insignificantly in 16-18-year-olds, and 1.483(1.128～1.949) in 13-18-year-olds. | screen time <=2h/d | 13 | moderate |
| 174 | Yunjuan Yang | 2015 | Chinese | Cross-sectional | Yunan | 9 -18 years | 18780 | 2-myopia | Low vision | TV; computer etc. | age, gender, family income, sleep hours, diary intake, egg intake, academic pressure, homework hours, physical activity | TV 1-2.99, >=3 vs. <1 h/d,myopia OR (95%CI) were 0.81 (0.75～0.87) and 0.65 (0.57～0.74). Computer 1-1.99, >=2 vs. <1 h/d was not associated with myopia. | TV <1h/d; computer <1h/d. | 15 | high |
| 175 | Yan Pang | 2009 | Chinese | Cross-sectional | n.a. | Grade 7-12 | 452 | 2-myopia | Low vision | TV, computer | gender, grade level, parent myopia, study hours, eading distance, reading while lying, after-school tutoring, physical activity, fruit and vegetables intake, picky eating. | ST >=2 h/d vs. <2 h/d: myopia OR (95%CI) was 2.14 (1.52～2.94). | screen time <2h/d | 10 | low |
| 176 | Dayong Qiu | 2019 | Chinese | Cross-sectional | Taizhou | Grades 6-12 | 2374 | 7-poor physical fitness | fail in fitness exam | screen time | gender, age, BMI, fitness level | weekday screen time >=2 vs. <2 h/d: fail rates in physical fitness exams OR(95%CI) was 1.43(1.22～1.69);  weekend screen time >=2 vs. <2 h/d: fail rates in physical fitness exams OR(95%CI) was 1.43(1.31～1.55). | weekday and weekend screen time <2h/d | 12 | moderate |
| 177 | Lan Cheng | 2016 | Chinese | Cross-sectional | Beijing | grades 3-5 | 1170 | 1-adiposity | overweight and obesity | TV, video, computer, games, iPad | gender, grade, MVPA | screen time >2h/d vs. <=2h/d, : overweight and obesity OR=2.45, 95%CI: 1.56～3.84, p<0.01 . | screen time <=2h/d | 14 | moderate |
| 178 | Yamei Li | 2017 | Chinese | Cross-sectional | Hunan Changsha | 7-17 years | 2028 | 4-cardiometabolic risks | dyslipidemia | TV, computer | gender, nationality, residency, family income, BMI, central obesity, fruit and vegetable intake, meat intake, sugary drink intake, physical activity, sleep hours | screen time >2 vs. <=2 h: dyslipidemia OR(95%CI) was 1.357(1.059～1.739)，p=0.016. | screen time <=2h/d | 14 | moderate |
| 179 | Jiling Fu | 2013 | Chinese | Cross-sectional | Shengyang, Xinxiang, Guangzhou, Chongqing | 11-19 years | 13817 | 3-psychobehavioral problems | self-injury | TV, video, MP4, DVD/VCD | location of residence, gender, grade level, smoking, drinking, age | weekday ST>2h/d vs. <=2h/d: self injury OR (95%CI) was 1.27 (1.06～1.54) in sub-mental health condition and null in healthy mental condition.   weekend ST>2h/d vs. <=2h/d: self injury OR (95%CI) was 1.37 (1.17～1.61) in sub-mental health condition and 1.39 (1.26～1.52)in healthy mental condition. | weekday and weekend screen time <=2h/d | 12 | moderate |
| 180 | Lizi Lin | 2018 | Chinese | Cross-sectional | Beijing | 7-11 years | 1788 | 1-adiposity | Obesity | TV, video, computer, games, iPad | gender, age, MVPA, homework hours, breakfast frequency, meal away from home frequency, sugary drink intake, fried food intake, fast food intake, parental education. | Screen time <2 vs. >=2h/d: obesity OR (95%CI) was 0.63 (0.46～0.87) . | screen time <2h/d | 16 | high |
| 181 | Xinyu Fan | 2004 | Chinese | Cross-sectional | Fujian Fuzhou | Grades 7-12 | 4611 | 3-psychobehavioral problems | mental health | Internet | none | Internet >14h/w vs. <=14h/w: emotional instability and anxienty (F = -2.387, p = 0.017), fatigue and lassitude (F = -2.782, p = 0.005). | none | 8 | low |
| 181 | Xinyu Fan | 2004 | Chinese | Cross-sectional | Fuzhou | Grades 7-12 | 4611 | 8-musculoskeletal injuries | lumbago, finger,wrist and arm pain | Internet | none | Internet >14h/w vs. <=14h/w: lumbago (t = -2.323, p = 0.02), finger,wrist and arm pain (t = -2.992, p = 0.003). | none | 8 | low |
| 182 | Xiaomei Lei | 2014 | Chinese | Cross-sectional | Shangxi Xi'an | Grades 1-6 | 1634 | 6-Sleep disorders | sleep disorders | bedtime TV | parental age, parental education, family incom, independent bedroom, homework load, family bedtime, parent smoking or drinking, family history of sleep disorders, parental emotional instability. | Bedtime TV >2h vs. <=2h: sleep disorders OR(95%CI) was 3.366(2.005～5.650). | bedtime TV <=2h | 13 | moderate |
| 183 | Xiaobo Su | 2019 | Chinese | Cross-sectional | Henan Zhengzhou | Grades 1-12 | 8926 | 2-myopia | Myopia | TV; cellphone or Internet | gender, grade level, location of residence, boarding school, reading posture, after-school tutoring, homework hours, outdoor activity. | TV (<1, 1-2, 2-3, >=3h/d ): myopia >0.05, not included in the model.  Non-TV ST (<1, 1-2, 2-3, >=3h/d): myopia >0.05, not included in the model. | TV <1h/d; non-TV screen time <1h/d | 13 | moderate |
| 184 | Zhirong Zhang | 2013 | Chinese | Cross-sectional | Shanxi, Guangzhou, Tibet | Grades 10-12 | 1472 | 9-sub health | Physical sub-health | Internet | Location, insufficient sleep time, academic burden, physical activity, alcohol drinking, picky eating | Daiy internet surfing time >1h vs. <=1h, OR (95%CI): 1.66 (1.07 ~ 2.57) for physical sub-health. | Internet <=1h/d | 11 | moderate |
| 185 | Xiaopeng Pang | 2014 | Chinese | Cohort | Rural Shanxi, Rural Gansu | 9-16 years | 18360 | 5-academic performances | changes in math exam scores | TV, computer, and cellphone | age, age ^2, sleep hours, math exam scores from last year, mental health scores, tutoring classes on weekend, myopia, parental education, living with parents, family income, etc. | Changes in math exam score as compared to last year: weekday TV 0-0.5, 0.5-1.5 >=1.5h vs. 0 (beta, p) were (-0.031, p<0.10), (0.027, p >0.10), (0.028, p >0.10); ; weekday computer 0-0.5, 0.5-1.5 >=1.5h vs. 0 (beta, p) were (-0.02, p >0.10), (-0.048, p >0.10), (-0.190, P< 0.01); weekday cellphone 0-0.5, 0.5-1.5 >=1.5h vs. 0 (beta, p) were (-0.057, p< 0.05), (-0.057, p >0.10), (-0.121, p >0.10). weekend TV 0-1, 1-3, >=3h vs. 0 (beta, p) were (0.080, p<0.01), (0.170, p <0.01), (0.152, p <0.01); ; weekend computer0-1, 1-3, >=3h vs. 0 (beta, p) were (-0.013, p >0.10), (-0.030, p >0.10), (-0.037, P> 0.1); weekend cellphone 0-1, 1-3, >=3h vs. 0 (beta, p) were (-0.061, p< 0.01), (-0.037, p >0.1), (-0.066, p >0.10). | weekday and weekend TV =0; weekday and weekend computer =0; weekday and weekend cellphone =0; | 11 | low |
| 186 | Gang Wu | 2011 | Chinese | Cross-sectional | Jiangsu Xuzhou | Grades 7-12 | 5899 | 5-academic performances | exam achievement | TV/video; internet; e-games | none | low vs. high achievement: hours spent on TV, internet, egames higher (x^2, p) were (6.6<0.05), (39.2,<0.01), and (48.9,<0.01). | none | 7 | low |
| 187 | Li Li | 2015 | Chinese | Cross-sectional | Shijiazhuang | Grades 7-12 | 5942 | 1-adiposity | Obesity | TV or video; e-games; Computer | none | TV/ video >=2h/d vs. < 2h/d: obesity rate χ2= 11.148, P=0.001) ;  E-game >=2h/d vs. < 2h/d: obesity rate χ2=25.123，P<0.001 ;  Computer >=2h/d vs. < 2h/d: obesity rate χ2=17.174，P<0.001). | none | 11 | moderate |
| 188 | Haihong Wu | 2019 | Chinese | Cross-sectional | Jiangsu Xuzhou | 6-14 years | 6220 | 1-adiposity | overweight and obesity | TV, internet, e-games | parental weight status, sweet food preference, sleep hours, breakfast intake, fast food intake, sugary drink intake | screen time ＞2 h vs <=2h: overweight and obesity OR (95%CI) was 1.18 (1.03～1.67) in primary school-aged boys, insignificant in primary school-aged girls, OR (95%CI) was 1.15 (1．04～1．96) in middle school boys, and 1.08 (0.88～1.34) in middle school girls . | screen time <=2h/d | 13 | moderate |
| 189 | Min Zhang | 2007 | Chinese | Cross-sectional | n.a. | 12-18 years | 1294 | 3-psychobehavioral problems | suicidal ideation | e-games | chronic diseases, maternal education, drinking, academic anxiety, interpersonal anxiety, impulsive tendencies | E-game (h/week): suicidal ideation OR (95%CI) was 1.11 (1.02～1.21). | unknown | 12 | moderate |
| 190 | Lan Cheng | 2016 | Chinese | Cross-sectional | 30 provinces | 9-11 years | 40692 | 1-adiposity | overweight and obesity | TV, computer, e-game | age, urban, province, diary intake, egg intake | screen time 1h/d vs.<= 1h/d: overweight and obesity OR (95%CI) was 1.13 (1.02～1.26) in girls and 1.02 (0.95～1.10) in boys. | screen time <=1h/d | 15 | high |
| 191 | Lianjian Yang | 2020 | Chinese | Cross-sectional | Chongqin | Grades 4-9 | 5306 | 1-adiposity | overweight and obesity | Screen time | gender, sleep hoursonly child, family income, parental weight status, parenting practices of limiting physical activity, child eating speed, breakfast intake, milk intake, sweets intake. | Screen time >2 vs. <=2h/d: overweight and obesity p > 0.05, not included in the model | screen time <=2h/d | 12 | moderate |
| 192 | Chuyu Fang | 2009 | Chinese | Case-control | Yanji | 9-13 years | 1004 | 1-adiposity | Obesity | TV | none | TV time >2h/d vs. <=2h/d: obesity rates between groups (X^2 = 25.67, p<0.001) | none | 9 | low |
| 193 | Zilong Zhang | 2013 | Chinese | Cross-sectional | 30 provinces and areas except for Tibet | 7-18 years | 215319 | 7-poor physical fitness | fail in fitness exam | TV, e-games, computer | gender ; location of residency ; family income; nutrition status; homework hours; physical activity | screen time >2 vs. <=2 h/d fail rates in physical fitness exams OR(95%CI) were 1.226(1.180～1.274). | screen time <=2h/d | 13 | moderate |
| 195 | WoWang | 2017 | Chinese | Cross-sectional | Chongqing | Grades 7-9 | 1889 | 3-psychobehavioral problems | self-conciousness | Internet | gender, age, paternal education, family relationships, academic performance, etc. | Internet and self-conciousness: b=-1.024, p<0.01. | none | 10 | low |
| 197 | Nan Wang | 2016 | Chinese | Cross-sectional | Beijing, Hunan, Ningxia | 5-18 years | 4164 | 1-adiposity | overweight and obesity | TV, video, computer, eletronic games, cellphone, tablet | age, gender, parental education, family income, dietary intake, physical activity | weekday screen time >2h, vs. <=2h/d, OR (95%CI) :1.10 ( 0.88～1.37) for overweight and obesity. Weekend screen time >2h/d vs. <=2h/d, OR (95%CI) : 1.31 (1.11～1.56) for overweight and obesity. | weekday and weekend screen time <=2h/d | 13 | moderate |
| 198 | Xiaofang Yang | 2017 | Chinese | Cross-sectional | Multiple areas | 10-18 years | 12221 | 7-poor physical fitness | Cardiopulmonary endurance | TV, e-games | location of residence, age , gender, active commuting type, physical activity, weekend activity | screen time >2h/d, 1-2 h/d vs. <=2 h/d: endurance (0=low level, high level=1) OR (95%CI) were 0.84(0.75～0.93 and 0.92 (0.84 ~ 1.00). | screen time <=2h/d | 13 | moderate |
| 199 | Xiaomei Lei | 2015 | Chinese | Cross-sectional | Shangxi Xi'an | Grades 1-6 | 2027 | 6-Sleep disorders | sleep disorders | TV | parental age, parental education, family incom, independent bedroom, homework load, family bedtime, parent smoking or drinking, family history of sleep disorders, parental emotional instability. | TV (unknown coding): sleep disorders OR(95%CI) was 3.362(2.001-5.648). | unclear | 11 | moderate |
| 200 | Na Yong | 2012 | Chinese | Cross-sectional | Chongqing | 8-13 years | 1417 | 6-Sleep disorders | sleep disorders | TV | age, paternal healthy behaviors, child health status. | weekday TV ＜1h/d, 1-2h/d, 2～3h/d, 3～4h/d, ＞4h/d vs. 0-1 h: sleep disorders OR(95%CI) were 1. 368(1.019～1.836), 1.328(0.953～1.851), 0.689(0.443～1.074), 0.894(0.238～3. 354), 0.861(0.166～4. 469). | weekday TV =0 | 13 | moderate |
| 201 | Huaiting Gu | 2019 | Chinese | Cross-sectional | Hubei Wuhan, Zhejiang Hangzhou, Shandong Jining | 5-15 years | 11668 | 3-psychobehavioral problems | dyslexia | TV; eletronic devices | gender, grade level, location of residence | dyslexia group vs. normal group: screen time p > 0.05. | none | 10 | low |
| 202 | Xiaohua Li | 2012 | Chinese | Cross-sectional | Zhejiang | 6-18 years | 2237 | 2-myopia | Myopia | TV, computer | age, age, parental myopia, rreading hours, sleep hours. | ST (unknown coding): myopia OR (95%CI) was 2.437 (1.362～6.284) . | none | 11 | moderate |
| 203 | Chuanxin Xu | 2010 | Chinese | Cross-sectional | Szechwan | Grades 4-9 | 2633 | 3-psychobehavioral problems | school adaptability | TV; Internet; | left-behind child, gender, grade level, academic performance, single child, familial income, relationship with parents, perceptions about social isolation, perceptions about social inequality | Internet (h/week) and school adapatability: p >0.05. TV (h/d) and school adapatability: b=-0.094, p<0.01. | none | 11 | moderate |
| 204 | Caiyun Feng | 2020 | Chinese | Cross-sectional | Yan'an | 7-18 years | 3503 | 2-myopia | Low vision | TV, e-games | reading distance, homework hours, outdoor time, sleep hours. | ST 1～2h, >2h vs. <1 h: myopia OR (95%CI) were 1.274 (1.052～1.541) and 2.393 (2.014～2.844). | screen time <1h/d | 14 | moderate |
| 205 | Xueyan Zhang | 2015 | Chinese | Cross-sectional | Jiangsu | Grades 3-5 | 2362 | 3-psychobehavioral problems | Depression | TV; e-games | gender, sleep quality, breakfast intake, out-of-school study hours, physical activity | E-games >=2h vs. <2 h/time: depression OR (95%CI) was 1.90 (1.16～3.10). | e-games <2 h/time | 13 | moderate |
| 206 | Yonghong Zeng | 2010 | Chinese | Cross-sectional | Guangdong Yangchun | Grades 1-6 | 1219 | 1-adiposity | overweight and obesity | TV | eating speed, appetite, vegetable preference, fried food intake, physical activity, parental weight status. | TV viewing time( <1 h, 1～ 2 h, >2 h, ordinal coding) : obesity OR (95%CI) was 1.423 (1.073～1.887) . | none | 11 | moderate |
| 207 | Haihong Wu | 2018 | Chinese | Cross-sectional | Jiangsu Xuzhou | Grades 1-6 | 3228 | 1-adiposity | overweight and obesity | TV, e-games, internet | parental weight status, sweet food preference, physical activity, sleep hours | screen time >2h/d vs. <=2h/d: overweight and obesity OR (95%CI) was 1.18 (1.03～1.67) in boys and 1.07 (0.79～1.44) in girls. | screen time <=2h/d | 15 | high |
| 208 | Hongbo Shi | 2010 | Chinese | Cross-sectional | Yuyao | 11-18 years | 2950 | 1-adiposity | overweight and obesity | TV; internet | parental BMI, feeding style, meal time, meat intake, transportation type, sleep hours, homework hours, physical activity, parental education. | TV > 2h/d was inversely associated with obesity rates (beta=-0.89,p =0.04) ;  Internet > 2h/d was positively associated with obesity rates (beta=0.41,p<0.01) . | none | 9 | low |
| 209 | Jianhua Li | 2018 | Chinese | Cross-sectional | Yunan | 6-15 years | 7214 | 2-myopia | Refractive error | eletronic devices | gender, age, nationality, familial myopia, homework hours, reading in bed. | ST 0.5-1, 1-2, 2-3, 3-4,>4 vs. < 0.5h/d: myopia OR (95%CI) were 1.10 (0.86～1.42) , 1.36 (1.01～1.82) , 2.07 (1.48～2.90) , 1.61 (1.05～2.47) , 1.70 (1.14～2.56) . | screen time <0.5h/d | 12 | moderate |
| 210 | Yanqun Yu | 2015 | Chinese | Cross-sectional | Hunan Changsha | 6-13 years | 1045 | 2-myopia | Myopia | eletronic devices | reading distance, parental myopia, homework hours, outdoor activity. | ST (0=＜"1 h/d", 1="1-2h/d", 2=">3h/d")，myopia OR(95%CI) was 6.124 (1.022～36.680) . | none | 10 | low |
| 211 | Liu X | 2019 | Chinese | Cross-sectional | Zhengzhou | 12-18 years | 1461 | 9-sub health | Physical and mental sub-health | Internet | single-parent family, stay-up late, academic burden, out-of-school physical ativity, dietary diversity, academic performance, academic burden, number of students, regular dietary pattern | Daily internet surfing time 1-2h, 2-3h, >3h vs. <=1h OR (95%CI): 1.23 (0.66～1.58), 0.78 (0.38～1.60), 2.42 (1.30～4.50) for physical and mental sub-health. | Internet <=1h/d | 13 | moderate |
| 212 | Rong Tian | 2017 | Chinese | Cross-sectional | 30 provinces and areas except for Tibet | 11-18 years | 1420708 | 7-poor physical fitness | Cardiopulmonary endurance | screen time | gender, location of residency, P.E. class hours, homework hours, breakfast intake, sleep hours, physical activity, weight status, parental support to physical activity | screen time >2 vs. <=2h: fail in endurance run OR(95%CI) were 1.16(1.14～1.19). | screen time <=2h/d | 14 | moderate |
| 213 | Liang Xu | 2017 | Chinese | Cross-sectional | Shengyang, Zhengzhou, Shenzhen | Grades 7-12 | 10270 | 8-musculoskeletal injuries | neck and shoulder discomforts | cellphone; TV; computer | gender, grade level, location of residence, physical activity, study pressure | cellphone >=40 min (sometimes, often vs. never): neck and shoulder discomforts OR(95%CI) were 1.32(1.15～1.52), 2.57(2.19～3.02), 4.66(3.95～5.49).  TV>=40 min (sometimes, often vs. never): neck and shoulder discomforts OR(95%CI) were 1.10(0.97～1.25), 1.90(1.62～2.22), 4.01(3.39～4.73). computer>=40 min (sometimes, often vs. never): neck and shoulder discomforts OR(95%CI) were 1.15(1.01～1.31), 2.07(1.76～2.43), 3.61(3.09~4.23). | none | 12 | moderate |
| 214 | Tingting Wang | 2014 | Chinese | Cross-sectional | Xinjiang Urumuqi | Grades 7-9 | 2462 | 2-myopia | Myopia | TV; computer | nationality, reading distance, unhealthy eye use habits, reading duration, sleep hours, outdoor time. | Computer 1 = "＜1 h/d"，2 = "1-2 h/d"，3 = " >2h/d: myopia OR (95%CI) was 1.158 (1.037 ～ 1.295) ;  TV 1 = "＜1 h/d"，2 = "1-2 h/d"，3 = " >2h/d": myopia OR (95%CI) was 0.910 (0.813 ～ 1.019) . | none | 12 | moderate |
| 215 | Xiaowei Chen | 2018 | Chinese | Cross-sectional | Shanghai | Grades 7-12 | 4966 | 5-academic performances | exam scores | non-TV screen time | gender, school type, location of residency. Family income, study pressure, parental education | non-TV screen time >1h/d vs. <=1h/d: attention, self-directed study, exam scores, relationship with schools OR(95% CI) was 1.59(1.21～2.10), 1.48(1.13～1.95), 1.44(1.09～1.89), 1.48(1.12～1.95). | non-TV screen time <=1h/d | 13 | moderate |
| 301 | Juan Zhang | 2012 | English | Cross-sectional | Jiangsu, Shandong, Henan, Hubei, Hunan, Guangxi | 6-18 years | 5497 | 1-adiposity | BMI | TV/video | Place of residence, Family income, Physical activity, Time spent on studying before and after school, Energy intake, fat% | Association between BMI and screen time was not significant in 6-11 years old male and 6-18 years old females, but was significantly positive among 12-18 years old males (b=0.076, p < 0.05). | none | 14 | moderate |
| 302 | Shang-yu yang | 2017 | English | Cross-sectional | Taiwan | 16-19 years | 302 | 8-musculoskeletal injuries | musculoskeletal discomfort | cellphone | Gender, Hours spent talking on the phone, Hours spent texting, Hours spent texting, Smartphone dependence, Degree of infuence | talking on the phone > 1-3h/d vs 0-1 h/d was associated with upper back pain and wrists/hands pain.  talking on the phone > 3h/d vs 0-1 h/d was associated with upper back pain and wrists/hands pain.  texting or using ancillary functions >3, 1-3 vs. <1 h/d were either not associated with discomforts in various parts or associated with lower risks of disconforts.  Smartphone use per weekend day were not associated with musculoskeletal discomforts in all six body parts. | talking, texting and weekend cellphone <1 h/d | 14 | moderate |
| 303 | Joseph T. F. Lau | 2017 | English | Cohort | Hong Kong | 12-16 years | 6954 | 3-psychobehavioral problems | Internet addiction | Internet | gender, school grade,  father’s and mother’s education, living arrangement, and place of birth | online entertainment per week 1-10, 11-20, >=21 hr/wk vs. 0 h/wk, incidence of internet addiction OR (95% CI) were 1.27 (0.80, 2.01), 2.00 (1.25, 3.22), 1.92 (1.20, 3.08). online communication per week 1-10, 11-20, >=21 hr/wk vs. 0 h/wk, incidence of internet addiction OR (95% CI) were 1.07 (0.83, 1.38), 1.36 (1.00, 1.85), 1.63 (1.21, 2.18). | Online entertainment 0 h/wk; online communication oh/week. | 14 | moderate |
| 304 | Wendy Yajun Huang | 2016 | English | Cohort | Hong Kong | Grades 1-3 | 672 | 1-adiposity | BMI | TV, electronic games, internet | Age, gender, and snacking habit of the child, parental education, parental BMI, and marital status | BMI at two-year follow-up were positively associated with replacing sedetnary time with screen time (b = 0.12, 0.04 ~ 0.21) . | none | 13 | moderate |
| 305 | Sze Pui Pamela Tin | 2012 | English | Cross-sectional | Hong Kong | Grades 1-9 | 70210 | 3-psychobehavioral problems | self-esteem | TV | age, gender, highest parental education level, occupational status, P4 BMI, frequency of extracurricular physical activity, frequency of computer or video game use, junk food, and fruit or vegetable consumption habits. | TV time 1-2, <2-4, >4 vs. < 1 hr, self-esteem OR (95% CI) 0.09 (0.02, 0.16), -0.27 (-0.34, -0.19), -0.91 (-1.00, -0.82). | TV < 1h/d | 17 | high |
| 306 | Hsin-Jen Tsai | 2009 | English | Cross-sectional | Taiwan | 11-12 years | 1329 | 1-adiposity | BMI | TV; e-games | none | TV watching hr/d, BMI correlation r in boys 0.063, NS, in girls r 0.089, p < 0.05.  Playing video game hr/d, BMI in boys and girls all NS. | none | 10 | low |
| 306 | Hsin-Jen Tsai | 2009 | English | Cross-sectional | Taiwan | 11-12 years | 1329 | 10-Miscellaneous | Respiratory symptoms | TV, electronic games | none | Correlations between TV watching (hr/d) and numbers of respiratory symptoms were r = 0.07, p > 0.05in boys and r = 0.104, p < 0.01 in girls. Correlation between video game (hr/d) and numbers of respiratory symptioms were not significant in boys and girls. | None | 10 | low |
| 307 | Chun-ying Lee | 2016 | English | Cross-sectional | Taiwan | 12-16 years | 2727 | 4-cardiometabolic risks | metabolic syndrone | TV, e-games, computer | Adolescent age, gender, ethnicity, residential area, total calorie intake, alcohol drinking and cigarette smoking, as well as parental overweight/obesity, diabetes mellitus and hypertension | Screen time >=3, 1.5-2.9 vs. <1.5h/d: metabolic syndrome OR (95%CI) were 2.1 (1.1-3.9) and 1.5 (0.8-2.8). After the additional adjustment of BMIz score, were 1.5 (0.6-3.6) and 1.5 (0.5-4.7). | ST <1.5 h/d | 16 | high |
| 308 | Po-Wen Ku | 2018 | English | Cohort | Taiwan | 7-12 years | 1431 | 2-myopia | Myopia | computer, Internet, e-games | age, parental educational levels, household income, urbanization, TV watching and outdoor leisure activities, and eye disorders | computer, internet, and video game 0.5-0.9, >=1, vs. <0.5 h/d, 2010-2013 incidence of myopia HR (95% CI) were 1.00 (0.76, 1.31) , 1.14 (0.89, 1.48) . | non-TV screen time <0.5 h/d | 18 | high |
| 309 | Yun Song | 2019 | English | Cross-sectional | Beijing, Shenyang, Chongqing, Zhengzhou | Grades 1-9 | 5959 | 3-psychobehavioral problems | emotional symptoms | TV, video, cellphone, other eletronic devices | gender, age, parental education, family economic level, parental smoking and any siblings | Screen time >2 vs. <=2 h/d: emotional symptoms OR (95% CI) were 1.39 (1.16–1.67). | screen time <=2h/d | 11 | moderate |
| 309 | Yun Song | 2019 | English | Cross-sectional | Beijing, Shenyang, Chongqing, Zhengzhou | Grades 1-9 | 5959 | 3-psychobehavioral problems | conduct problems, hyperactivity problems, prosocial behavior, and total difficulties | TV, video, cellphone, other eletronic devices | gender, age, parental education, family economic level, parental smoking and any siblings | Screen time >2 vs. <=2 h/d, conduct problems, hyperactivity problems, prosocial behavior and total difficulties OR (95% CI) were 1.77 (1.48–2.12), 1.60 (1.32–1.94), 1.42 (1.20–1.67), and 1.65 (1.41–1.93). | screen time <=2h/d | 11 | moderate |
| 310 | Hsin-Jen Tsai | 2007 | English | Cross-sectional | Taiwan | 11-12 years | 2218 | 1-adiposity | overweight and obesity | TV | residential districts, gender and physician-diagnosed allergy | TV watching 1-2, >=3 vs.<1 h/d: overweight OR (95%CI) was 1.22 (0.88, 1.69), 1.44 (1.01–2.06); obesity OR (95%CI) were 1.02 (0.73, 1.42) , 1.49 (1.05, 2.11) . | TV <1h/d | 9 | low |
| 310 | Hsin-Jen Tsai | 2007 | English | Cross-sectional | Taiwan | 11-12 years | 2218 | 10-Miscellaneous | Respiratory symptoms | TV | residential districts, gender and physician-diagnosed allergy | TV watching 1-2 vs.<1 hr/d, OR: not significant for persistent cough, chest tightness, wheezing with cold, wheezing without cold, dyspnea , exercise-induced wheezing, exercise induced cough. TV watching >=3 vs.<1 hr/d, OR (95% CI): 1.62 (1.20–2.18) for persistent cough, 1.56 (1.15–2.13) for chest tightness, 1.13 (0.82–1.57) for wheezing with cold, 1.90 (1.27–2.85) for wheezing without cold, 1.48 (1.08–2.02) for dyspnea asociated with wheezing, 1.17 (0.91–1.50) for exercise-induced wheezing, and 1.42 (1.11–1.81) for exercise induced cough. | TV <1h/d | 9 | low |
| 311 | Wei Zheng | 2016 | English | Cross-sectional | Beijing, Guangzhou, Chengdu, Shengyang, Suzhou, Zhengzhou, Hebei rural area | Grades 1-6 | 770 | 4-cardiometabolic risks | dyslipidemia | TV, video, computer, cellphone | age, total energy intake per day, family income,and PA | screen time any item >=1.0 h/d vs. all items < 1.0 h/d, dyslipidemia OR (95% CI) in all children, boys, and girls were 2.11 (1.11, 3.99), 3.04(1.24, 7.45), and 1.83 (0.64, 5.29). | screen time all items < 1 h/d | 14 | moderate |
| 312 | Xin Huang | 2019 | English | Cross-sectional | Zhejiang | 8-19 years | 1040 | 5-academic performances | exam scores | TV, video, cellphone, tabletcomputer, computer | age, school,grade, and class | screen based sedentary behaviors, academic performance in girls b = -1.19, p < 0.001, in boys b = -0.82, p < 0.001. | none | 12 | moderate |
| 312 | Xin Huang | 2019 | English | Cross-sectional | Zhejiang | 8-19 years | 1040 | 7-poor physical fitness | physical fitness | TV, video, cellphone, tabletcomputer, computer | age, school,grade, and class | screen based sedentary behaviors, physical fitness in grils b=-0.46, p < 0.05, in boys b= -0.22, p > 0.05. | none | 12 | moderate |
| 313 | Lian Tong | 2016 | English | Cross-sectional | Shanghai | 9-13 years | 785 | 3-psychobehavioral problems | ADHD | TV, cellphone, computer | children's gender, age, parents' education level, parents' age, annual household income | screen time, ADHD b=0.04, p >0.05. | none | 11 | moderate |
| 314 | Jie-yun Song | 2017 | English | Cross-sectional | Beijing | 7-18 years | 2179 | 1-adiposity | BMI | TV, video, computer | age and gender | BMI was positively associated with Screen time ( >=2 vs. < 2h/d) among those with rs12970134 variant (b=0.74, p =0.048) . | none | 12 | moderate |
| 315 | Hsien-yu Fan | 2018 | English | Cohort | Taiwan | 6-11 years | 5572 | 1-adiposity | BMI | TV, computer, eletronic games | Parental education and family income. | Compared with persistently healthy weight group, late-onset overweight or obesity, persisitent overweight or obesity, declining BMI group were all more likely to have greater screen time at baseline, OR (95% CI) : 1.05 (1.01, 1.08) , 1.07 (1.04, 1.10) , 1.10 (1.02, 1.18) . | none | 10 | low |
| 316 | Zhi Shan | 2013 | English | Cross-sectional | Shanghai | 15-19 years | 3016 | 8-musculoskeletal injuries | neck/shoulder pain, lower back pain | computer, cellphone, tabletcomputer | gender and age | cellphone 1-1.5, 1.5-2, >2 vs. < 1h/d, neck/shoulder pain OR (95% CI) were 1.17 (0.97, 1.42), 0.90(0.69, 1.16), 1.49(1.20-1.86); lower back pain OR (95% CI) were 1.44 (1.18, 1.77), 1.22(0.93, 1.60), 1.84 (1.46, 2.32).  computer 1-1.5, 1.5-2, >2 vs. <1h/d, neck/shoulder pain OR (95% CI) were not significant.  tablet 1-1.5, 1.5-2, >2 vs. <1h/d, neck/shoulder pain OR (95% CI) were not significant. | cellphone <1h/d; computer <1h/d; tablet <1h/d; | 10 | low |
| 317 | MA guan-sheng | 2002 | English | Cross-sectional | Guangzhou, Shanghai, Jinan, Harbin | 4-16 years | 9356 | 1-adiposity | Obesity | TV | gender, age, region, housing situations, income, parents' education levels, breakfast frequency, fast food consumption, desired body size. | TV time 1-2, 2-3, >3 vs. <1 h/d: obesity OR (95%) were 1.120 (0.949, 1.279) , 1.253 (1.031, 1.523) , 1.398 (1.075, 1.819) . | TV <1h/d | 12 | moderate |
| 318 | Shengxin Liu | 2019 | English | Cross-sectional | Tianjin | 6-14 years | 566 | 2-myopia | Myopia | TV; tablets; computer; cellphone | age, gender, BMI,monthly family income, parental myopia, time spent out doors, time spent reading and writing, and daily sleep duration | Time spent using smart phones, myopia OR (95% CI) was 0.90 (0.57, 2.28) , refractive error b = -0.07, p = 0.042; axial length b= 0.10, p =0.006. Time spent using tablets, myopia OR (95% CI) was 1.40 (0.84, 2.37) ; refractive error b = -0.19 p = 0.17; axial length b= -0.03, p =0.45. Time spent using computers, myopia OR (95% CI) was 1.41 (0.84, 2.37) ; refractive error b = -0.08, p = 0.018; axial length b= 0.10, p =0.002. Time spent watching television, myopia OR (95% CI) was 0.89 (0.61, 1.28) ; refractive error b = 0.02, p = 0.50; axial length b= -0.03, p =0.45. | none | 16 | high |
| 319 | Hongyu Guan | 2019 | English | Cross-sectional | Northwest China | 9-12 years | 19934 | 2-myopia | Myopia | TV; computer; cellphone | Grade, Age, gender, Family Wealth, parental migrant status, parental education, child’s residence | time using computers 1-30min, 31-60min, >60 min vs. 0 min， mypopia beta (95%CI) were 0.017 (-0.097-0.131) , 0.305 (0.141-0.468) , 0.032 (-0.161-0.226) . time using smartphones 1-30min, 31-60min, >60 min vs. 0 min， mypopia all insignificant;  TV viewing time 1-30min, 31-60min, >60 min vs. 0 min， mypopia all insignificant; | computer = 0. TV = 0. cellphone = 0. | 15 | high |
| 320 | Li-Na Chou | 2017 | English | Cross-sectional | Taiwan | Grade 5 | 2351 | 1-adiposity | overweight and obesity | TV; Computer, e-games | gender, residential area, physical activity levels, sedentary behaviors, dietary habits, and perceived body shape on BMI | TV 2-3h/d vs. none: abnormal BMI OR (95%CI) 1.510 (1.025-2.226) ;  Holiday video games and computer 1h, 2-3h vs. none: abnormal BMI OR (95%CI) were 0.747 (0.596-0.935) , 0.648 (0.482-0.871) . | TV none; Holiday video games and computer none | 15 | high |
| 321 | Yi-Ching Lin | 2019 | English | Cross-sectional | Taiwan | 7-12 years | 1031 | 1-adiposity | overweight | TV, computer, cellphone | grade and gender | screen time >=2h/d vs. <2h/d: overweight OR (95%CI) was 5.68 (3.86-8.38) . | screen time <2h/d | 14 | moderate |
| 322 | FANG-yI TSENG | 2014 | English | Cross-sectional | Taiwan | 13-18 years | 391 | 3-psychobehavioral problems | Self-injury | Internet | Grade, Family constitution type, Depression score, Web, Family support score, Enough perceived friends, No. of friends to confide in, No. of friends available to seek help from | Time spent on the Internet >=1 hr vs. <1 hr, thoughts of nonsuicidal self-injury OR (95%CI) in boys was not sigificant, girls were 3.56 (1.18–10.70), no signficiant associations with suicidal ideation, suicide plan and nonsuicidal self-injury.  Time spent on online chatting > 0.5 hr vs. <=0.5 hr, no significant associations. | internet <1h/d | 12 | moderate |
| 323 | Liangli Li | 2015 | English | Cross-sectional | Shanghai | 6-12 years | 2400 | 1-adiposity | Obesity | TV, video, Computer | age,gender, household income, modes of travel to/from school, snacks/screen time + SSB + physical activity | screen time <=2, 2-3 vs. >3 hrs/d: obesity OR (95% CI) were 0.37 (0.21-0.66) , 0.38 (0.18-0.79) ; overweight and obesity OR (95% CI) were 0.63 (0.39, 1.03) , 0.81 (0.45, 1.44) . | screen time <2h/d | 12 | moderate |
| 324 | Shuo Wang | 2016 | English | Case-control | Beijing | 7-18 years | 1027 | 4-cardiometabolic risks | non-alcoholic fatty liver disease | TV, e-games | age and gender | Screen time >=2 h/d vs. < 2h/d, NAFLD OR (95%CI)was 1.64 (1.14, 2.36). | screen time <2h/d | 13 | moderate |
| 325 | Yiing Mei Liou | 2010 | English | Cross-sectional | Taiwan | 13-16 years | 8640 | 1-adiposity | Obesity | TV; Computer; internet; e-games | Physical activity, Sleep, parental obesity, eating night snack | weekday TV >=2hr/d, obesity b (95%CI) in males was 1.432 (1.107,1.917) , in females was 1.769 (1.101, 2.844) ;  weekend TV >=2hr/d, obesity not significant;  weekdays and weekends non-TV screen time: obesity not significant. | none | 16 | high |
| 326 | Qiying Song | 2019 | English | Case-control | Beijing | 6-18 years | 1404 | 1-adiposity | obesity | TV, computer, electronic games | gender, age, and age^2 | Screen time >=2 vs. < 2h/d, obesity risk among those with rs2943650 variant OR (95%CI) was 1.71 (0.44, 6.62) . | screen time <2h/d | 16 | moderate |
| 327 | Carrie E. Waller | 2003 | English | Cross-sectional | Nine provinces | 6-11 years | 1385 | 1-adiposity | overweight | TV, video | none | Overweight vs. nonoverweight, television viewing time 3.7 ± 4.3 vs. 5.5 ± 5.1 hrs/wk, p < 0.02; video no significant difference. | none | 12 | moderate |
| 328 | Jiao Fang | 2018 | English | Cross-sectional | Anhui | 7-11 years | 997 | 1-adiposity | BMI | Screen time | gender, age and self‐perceived socioeconomic status | BMI was inversely associated with lower screen time (< 2h/d vs. >=2h/d) : b= -0.84 , 95% CI: -1.44, -0.25. | none | 14 | moderate |
| 329 | Jyu-Lin Chen | 2007 | English | Cohort | Taiwan | 7-8 years | 307 | 1-adiposity | BMI | TV, video, video games | Baseline BMI, PACER test results, sit-and-reach  test results, sit-up test results, physical activity time,dietary intake, and maternal overweight status | BMI at one-year follow-up was positively associated with television and computer time ( b=0.156, p =0.059, sr^2 = 0.01) . | none | 13 | moderate |
| 330 | L Guo | 2016 | English | Cross-sectional | Guangzhou | Grades 1-9 | 3055 | 2-myopia | Myopia | TV; computer; e-games | age, grade level, ethnicity, daily hours of reading or studying for school assignments, daily hours of reading for pleasure, weekly hours of watching television, weekly hours of using computer, weekly hours of playing electronics, distance of reading, distance of watching television, and number of parents with myopia | Weekly hours of watching television <2, 2-4, >4 h vs. none, myopia OR (95% CI) in boys not significant, in girls were 1.77 (1.21, 2.58) , 1.94 (1.29, 2.90) , 2.60 (1.53, 4.41) . Weekly hours of using computer <2, 2-4, >4 h vs. none, myopia not significant;  Weekly hours of playing electronics, myopia not significant. | computer = 0. TV = 0. egames = 0. | 15 | high |
| 331 | Xiaohua Li | 2010 | English | Case-control | Changsha, Shenzhen | 6-9 years | 418 | 1-adiposity | Obesity | TV | paternal obesity, maternal obesity,maternal weight gain during pregnancy,birth weight,feeding mode,time of adding meat,frequency of having western snacks,duration of a meal,duration of watching TV,duration of playing outside | TV >=2h/d vs. < 2h/d: obesity OR (95%CI) was 2.35 (1.01-5.47) . | TV <2h/d | 11 | low |
| 332 | Xiaoqing Yi | 2012 | English | Case-control | Xi'an | 7-18 years | 516 | 1-adiposity | obesity | TV, computer, e-game | Sleeping time, Parental overweight, Family history of diabetes, High maternal education | Watching TV, playing video games and using computers (h/d) , obesity OR (95% CI) was 1.564 (1.133–2.159) . | none | 17 | high |
| 333 | Zhihao Xie | 2020 | English | Cross-sectional | Chongqing | 7-13 years | 997 | 2-myopia | Myopia | TV, e-games | age, gender, parental myopia,outdoor time, homework time | TV time 1-2h, 2-3 h, >=3h vs. <1h/d, myopia OR (95% CI) were 1.471 (0.998, 2.169) , 1.027 (0.577, 1.828) , 2.106 (1.200, 3.697) . Electronics time > 1h vs. <=1 h/d, myopia OR (95% CI) was 2.983 (2.088, 4.262) . | TV <1h/d;  Electronics < 1h/d | 13 | moderate |
| 334 | Ting Zhang | 2016 | English | Cross-sectional | Guangzhou, Shanghai, Jinan, Harbin | 7-12 years | 3766 | 1-adiposity | overweight and obesity | TV; Computer | age, gender, only child or not, paternal and maternal educational level, paternal and maternal occupation and monthly household income | Viewing television hr/d, overweight and obesity OR (95% CI) were 1.02 (0.84, 1.23) , 1.10 (0.91, 1.32) ;  Using a computers hr/d, overweight and obesity OR (95% CI) were 1.01 (0.81, 1.26) , 1.04 (0.84, 1.30) . | none | 14 | moderate |
| 335 | Jianghong Liu | 2019 | English | Cross-sectional | Shandong | Grades 7-11 | 11831 | 3-psychobehavioral problems | Depression | cellphone | age, gender, chronic disease, smoking, alcohol use, sleep duration, insomnia, school, economic status, interparental relationship, father education, and occupation) | Cellphone use 1-2 h/d, >=2h/d vs. <1h/d on weekdays, depression OR (95% CI) were 1.16 (0.95, 1.41), 1.78 (1.48, 2.15).   Cellphone use 2-4 h/d, 4-5h/d, >=5h/d vs. <2h/d on weekend days, depression OR (95% CI) were 1.04 (0.88, 1.22), 1.11 (0.85, 1.43), 1.67 (1.41–1.98). | weekday and weekend cellphone <1h/d | 10 | low |
| 336 | Hsiu-Mei Huang | 2013 | English | Case-control | Taiwan | 9-10 years | 550 | 1-adiposity | Obesity, central obesity | TV; Computer; e-games | unknown | ST >2h/d vs. <=2h/d, obesity and central obesity ORs (95%CI) were 1.57 (1.11, 2.24) and 1.92 (1.35, 2.73) .  TV >2h/d vs. <=2h/d, obesity and central obesity ORs (95%CI) were 1.59 (1.06, 2.41) and 1.64 (1.09, 2.48) .  Computer >1h/d vs. <=1h/d, obesity and central obesity were not significant. | screen time <=2h/d; TV <=2h/d; Computer <=1h/d | 14 | moderate |
| 337 | Runsen Chen | 2020 | English | Cross-sectional | Shenzhen | Grades 7-12 | 1835 | 3-psychobehavioral problems | self-injury | cellphone | gender, father education background, academic pressure for attempted suicide, single child, father education background, mother education background and academic pressure for self-harm | Cellphone use (<1 h, 1–2 h, 2–3 h, 3–4 h, >4 h/d, ordinal coding), suicidal ideations, suicidal planning, attempted suicide and self-harm OR (95% CI) were 1.066 (1.014, 1.121), 1.075 (1, 1.155), 1.184 (1.098, 1.276), 1.067 (0.998, 1.141). | none | 14 | moderate |
| 338 | Cheng-Fang yen | 2010 | English | Cross-sectional | Taiwan | Middle schoolers including professional middel schoolers | 9278 | 1-adiposity | BMI | TV; internet; cellphone | gender, age, residential back ground, paternal and maternal education and exercise leve | High television viewing (>=2 hr/d) ,BMI b=0.036, p <0.001. High weekly internet use (>=20 hr/wk) , BMI b=0.019, p >0.05. Highi daily cellphone use (>=1 hr/d) , BMI not significant. | none | 12 | moderate |
| 339 | Hui Cao | 2011 | English | Cross-sectional | Anhui Bengbu | 11-16 years | 5003 | 3-psychobehavioral problems | depressive symptoms, anxiety symptoms and school life dissatisfaction | TV, computer | gender, grade, family type, perceived socioeconomic status, BMI weight status (obesity), fruit and vegetable intake and fizzy drinks intake | Screen time > 2 h/d vs. <=2h/d, depressive symptoms, anxiety symptoms and school life dissatisfaction OR (95% CI) were 1.52 (1.31–1.76), 1.36 (1.18–1.57), 2.07 (1.79–2.40). | screen time <=2h/d | 12 | moderate |
| 340 | Carol Strong | 2018 | English | Cohort | Taiwan | Grades 7-9 | 3795 | 3-psychobehavioral problems | depressive symptoms, school integration | Internet | gender, Parental Education, Urbanization Level of Residence, and Academic Achievement | changes of internet use was positively associated with depressive symptoms at wave 4 b=0.31, p < 0.05, but was not associated with school integration. | none | 14 | moderate |
| 341 | Feizhou Zheng | 2014 | English | Cross-sectional | southwestern China | 12-20 years | 7102 | 3-psychobehavioral problems | inattention | cellphone | age, gender, area of residence (urban/rural) and whether living close to mobile base stations | cellphone use for entertainment > 60, 21-60 vs. 0-20 min/d, inattention OR (95%CI) were 1.87 (1.28-2.73), 1.82 (1.28-2.59). | cellphone 0-20 min/d | 11 | moderate |
| 342 | Weina Liu, MS | 2019 | English | Cross-sectional | Jiangsu | Middle schoolers, college students, graduate students | 46611 | 1-adiposity | BMI | TV, e-games, internet | Gender,age,Mother’s education,Allowance per month,Geographic region,Grade, | BMI was postively associated with screen time( b = 0.022, p < 0.001). | none | 10 | low |
| 343 | H-Q. Zhou | 2011 | English | Cross-sectional | Shanghai | 12-18 years | 1221 | 6-Sleep disorders | sleep quality | TV | age,gender, family income, family structure and parents’ educational levels | television viewing during weekdays(<1, 1-2, 2-3,3-4, >4), poor sleep quality OR (95% CI) was 1.56 (1.36–1.71);  television viewing on weekend(<1, 1-2, 2-3,3-4, >4), poor sleep quality not significant. | none | 14 | moderate |
| 344 | Hong Ren | 2016 | English | Cross-sectional | Beijing, Guangzhou, Hefei, Shanghai, Xi'an | n.a. | 5032 | 1-adiposity | obesity | TV, electronic games | gender and grade level | BMIz score was positively associated with weekday screen time (b=0.07, p < 0.03) but not with weekend screen time ( b=0.021, p > 0.05) . BMIz score was positively associated with weekday screen time in girls (b = 0.09, p = 0.05) but not in boys. | none | 11 | moderate |
| 345 | Xianchen Liu | 2020 | English | Cohort | three counties of Shandong Province | n.a. | 856 | 5-academic performances | exam scores | cellphone | gender, age, chronic disease, smoking, drinking, sleep duration, anxiety, depression, family economic status, interparental relationship, parents' education and occupation. | Weekday cellphone use >=2, 1-2, vs. < 1 hrs/d, Chinese, math and english grades trend b=-2.5, -3.17, -3.22, all p <0.0001. weekend cellphone use >=2, 1-2, vs. < 1 hrs/d, Chinese, math and english grades trend b=-0.32, p=0.081, b=-0.64,p=0.003, b=-0.58, p=0.004. | weekday and weekend cellphone use < 1 h/d | 13 | moderate |
| 346 | Si-Tong Chen | 2020 | English | Cross-sectional | Nanjing | Grades 4-12 | 2614 | 1-adiposity | overweight, obesity | TV, computer, e-game, eletronic devices | gender, urban/urban, family composition, family income, parental education level | non-significant associations were discovered in the screen time with weight status | none | 15 | high |
| 347 | Phoenix K.H. Mo | 2018 | English | Cross-sectional | Hong Kong | 10-17 years | 862 | 3-psychobehavioral problems | emotion dysregulation | Internet | none | Emotion dysregulation and number of hours spent online per week had a significant positive correlation (r = 0.18, p < 0.01). | none | 5 | low |
| 348 | Tingting Gao | 2020 | English | Cross-sectional | Changchun | Grades 10-12 | 2272 | 3-psychobehavioral problems | mood symptoms | Internet | Gender, School grade, Academic class, sleep deprivation on weekdays, use of internet gaming, moter-child relationship, father-child relationship | internet use on weekdays >30 min vs. 0-30 min, mood symptoms OR (95%CI) was 1.31 (1.04, 1.64);  internet use on weekends 120-239 min, >=240 min. vs. 0-119 min, mood symptoms not significant. | weekday internet <0.5h/d, weekend internet <2h/d | 9 | low |
| 349 | Shu Hu | 2020 | English | Cross-sectional | China | Grades 7-9 | 19487 | 3-psychobehavioral problems | Depression | TV, Internet, e-games | Gender, Age, Only child status, Hukou status, Parents’ migration status, parents’ employment status, Subjective economic status, Caregiver’s educational expectation, self-rated health status | screen time, depressive symptoms b = 0.005, all p < 0.001. | none | 11 | moderate |
| 349 | Shu Hu | 2020 | English | Cross-sectional | China | Grades 7-9 | 19487 | 5-academic performances | academic performance | TV, internet, e-games | Gender,Age,Only child status,Hukou status,Parents’ migration status,parents’ employment status,Subjective economic status,Caregiver’s educational expectation,self-rated health status | screen time, academic performance b = -0.084, cognitive development b = -0.052, educational aspiration b = -0.020, confidence in the future b= -0.003. | none | 11 | moderate |
| 350 | Bei Lu | 2009 | English | Cross-sectional | Rural Shantou, Guangdong | Grades 7-8 | 1892 | 2-myopia | Myopia | TV, e-games, computer | age, gender, parent's highest education, homework, personal reading, outdoor activities. | watching television (diopter-hours/wk) , myopia: 1.41 (0.82, 2.41) , p = 0.21;  playing video games/computer use (diopter-hours/wk) , myopic: NE. | none | 17 | high |
